# Bulk MgB_2_ Superconducting Materials: Technology, Properties, and Applications

**DOI:** 10.3390/ma17112787

**Published:** 2024-06-06

**Authors:** Tetiana Prikhna, Vladimir Sokolovsky, Viktor Moshchil

**Affiliations:** 1 V. Bakul Institute for Superhard Materials, National Academy of Sciences of Ukraine, 2, Avtozavodska Str., 07074 Kyiv, Ukraine; prikhna@ukr.net (T.P.); vik_ism@ukr.net (V.M.); 2 Institut de Ciencia de Materials de Barcelona, Spanish National Research Council (CSIC), Campus UAB, 08193 Bellaterra, Spain; 3 Leibniz-Institut für Festkörper-und Werkstoffforschung Dresden e.V., Helmholtzstraße 20, 01069 Dresden, Germany; 4 Faculty of Engineering and Information Sciences, School of Physics, University of Wollongong, Northfields Ave, Wollongong, NSW 2522, Australia; 5 Physics Department, Ben-Gurion University of the Negev, Beer-Sheva 8410501, Israel

**Keywords:** magnesium diboride bulk superconductors, superconducting properties, structural study, pinning, effect of impurity oxygen

## Abstract

The intensive development of hydrogen technologies has made very promising applications of one of the cheapest and easily produced bulk MgB_2_-based superconductors. These materials are capable of operating effectively at liquid hydrogen temperatures (around 20 K) and are used as elements in various devices, such as magnets, magnetic bearings, fault current limiters, electrical motors, and generators. These applications require mechanically and chemically stable materials with high superconducting characteristics. This review considers the results of superconducting and structural property studies of MgB_2_-based bulk materials prepared under different pressure–temperature conditions using different promising methods: hot pressing (30 MPa), spark plasma sintering (16–96 MPa), and high quasi-hydrostatic pressures (2 GPa). Much attention has been paid to the study of the correlation between the manufacturing pressure–temperature conditions and superconducting characteristics. The influence of the amount and distribution of oxygen impurity and an excess of boron on superconducting characteristics is analyzed. The dependence of superconducting characteristics on the various additions and changes in material structure caused by these additions are discussed. It is shown that different production conditions and additions improve the superconducting MgB_2_ bulk properties for various ranges of temperature and magnetic fields, and the optimal technology may be selected according to the application requirements. We briefly discuss the possible applications of MgB_2_ superconductors in devices, such as fault current limiters and electric machines.

## 1. Introduction

Modern progress in the development of new superconducting materials has brought the manufacturing industry to the stage of real applications. The most promising for wide application in various fields are MgB_2_ superconductors and high-temperature superconductors (HTS) based on rare-earth barium copper oxides and bismuth strontium calcium copper oxides [1,2,3,4,5]. This group may soon be supplemented by a class of iron-based superconducting compounds (or FeSC) [1], for which the production technologies are being intensively developed. Of all the mentioned materials, MgB_2_-based superconductors are the cheapest and most easily prepared for magnetic applications. The high level of superconducting characteristics of MgB_2_, which are very important for applications, such as critical current density, and upper critical and trapped magnetic fields, can be achieved in a polycrystalline structure due to the absence of the weak–link problem at grain boundaries [6]. The last represents the main drawback of HTS. This distinguishes magnesium diboride from HTS, which must be texturized or epitaxially grown to achieve high superconducting properties. In addition, the deviation of stoichiometry from MgB_2_ to a sufficiently high degree is not an obstacle to achieving a high level of superconducting characteristics [7,8,9,10,11]. The temperature of the superconducting transition of the MgB_2_ compound is about 39 K, depending on the isotope composition [12]. The critical temperature is lower than that of HTS, but is high enough for application in cryogenics devices in which liquid hydrogen (boiling temperature 20 K) and cryocoolers can be used for cooling.

Liquid hydrogen, when it is produced using renewable sources, is a promising green fuel with zero carbon emissions. Its high energy density makes it an ideal fuel source for transport and industry feedstock [13,14,15]. Since liquid hydrogen is more compact than hydrogen gas, its efficient storage and transportation are of great interest.

All the above-noted research has increased interest in the development of various superconducting devices that work at liquid hydrogen temperatures, such as motors, generators, transformers, pumps, magnetic bearings, fault current limiters, energy storage devices, transmission cables, magnets, resonators, MAGLEV transport, screens from electromagnetic irradiation, etc. [16,17,18,19,20,21,22,23,24]. The construction of superconducting MgB_2_ devices could contribute to further progress in hydrogen energy.

The properties of magnesium diboride compounds differ somewhat from those of other superconductors. Some of these differences stem from the MgB_2_ structure. The compounds possess a hexagonal crystal structure, hP3, with a space group of P6/mmm. The lattice parameters are *a* = *b* = 3.084 ± 0.001 Å and *c* = 3.522 ± 0.002 Å [25]. Their layered stacking consists of alternating Mg and B layers [26]. The bulk density according to Wikipedia is 2.57 g/cm^3^ and according to [25] it is 2.63 g/cm^3^, the melting point is 830 °C. The materials have a bulk modulus of about 172 GPa. The unit cell of MgB_2_ crystals demonstrates an anisotropic compressibility: the compressibility along the *c* axis is higher than that along the *a* and *b* axes [27]. Bulk MgB_2_ materials demonstrate isotropic characteristics, e.g., critical current density.

Many publications have been devoted to the investigation of the various properties of MgB_2_ superconductors and their theoretical considerations (e.g., [19,28,29,30,31,32,33,34,35,36,37,38,39,40,41,42,43,44,45,46,47,48,49,50,51,52,53,54,55,56] and the references therein). MgB_2_’s properties are considered more similar to metal than to those of HTS [28]. In this review, we limit ourselves to the analyses of the dependences of superconducting properties on the technology conditions and additions. Here, some theoretical results are noted only.

The theoretical understanding of the properties of MgB_2_ superconductors has nearly been achieved by the consideration of two energy gaps. The measured and estimated gaps of the π- and σ-bands of the electrons of MgB_2_ are typically around 2 meV and 6.5 meV, respectively [29,34,35,38,39]. In [39], it was noted, that these gaps can vary in the ranges of 1–4 meV and 5.5–10 meV.

Due to their comparatively high coherence lengths (ξ_ab_(*T* = 0) = 3.7–12 nm and ξ_c_(*T* = 0) = 1.6–3.6 nm) [12], the grain boundaries in MgB_2_ materials are not the obstacles for tunneling superconducting currents. The grain boundaries can be efficient pinning centers [12]. MgB_2_ is a type II superconductor with estimated values of the London penetration depth λ(0 K) = 125–140 nm and Ginzburg–Landau parameter *κ*(0) = 26 [12,37]. The authors of [36] estimated the low-temperature penetration depth of a MgB_2_ film as λ_c_ = 40 and λ_ab_ = 140 nm.

Recently, the electron localization functions and their isosurfaces were studied in [11].

Despite the structure of a unit cell of MgB_2_, it is simple and this compound nominally contains only two elements—Mg and B, the structure of MgB_2_-based materials can be complicated due to the presence of an admixture of oxygen, carbon, and even hydrogen and an inhomogeneous boron distribution. An oxygen impurity is usually present in a large amount (compared to carbon) even in materials prepared under ‘clean’ conditions in protective atmospheres. This is a result of the high affinity of magnesium toward oxygen. The carbon and hydrogen admixtures in MgB_2_ materials can appear due to their presence in the initial boron powder or absorption from atmosphere.

The methods for MgB_2_ bulk material preparation, which allow achievement of a relatively high level of superconducting characteristics, that make these materials suitable for practical application, are the following: (1) infiltration method (Inf.) [57], (2) sintering in Ar under atmospheric pressure (PL), (3) hot pressing (HotP), (4) spark plasma sintering (SPS) [20,58,59], (5) hot isostatic pressing (HIP) [60,61], (6) field-assisted sintering technique (FAST) [62,63], (7) shock consolidation method [64], and (8) high quasi-hydrostatic pressing (HP) [20,63] or ultra-high-pressure-assisted sintering [65].

Among the dozens of studied additions to MgB_2_, the ones that are the most effective from the point of view of an increase in the critical current density are carbon, carbon-containing compounds, silicon carbide, titanium, tantalum, zirconium, and compounds containing these metals [38,39,40,41,42,43,44,45,46,47,48,49,50,51,52,53,54,55,56,57,58,59,60,61,62,63,64,65,66,67,68,69,70,71,72,73,74,75,76,77,78,79,80,81,82,83,84,85,86,87,88,89]. Relatively recently, in the literature [78,90,91,92,93,94,95,96,97,98,99,100], there has been information about the positive effects on the superconducting characteristics of MgB_2_-based materials of Si_3_N_4_, hexagonal, cubic BN (boron nitride), NbB_2_, NbTi, Ni-Co-B, Rb_2_CO_3_ and Cs_2_CO_3_ additions and conflicting results have been presented about the effects of the following oxygen-containing additions: Dy_2_O_3_, SnO_2_, Sn-O, Ti-O.

The present overview is related to the preparation of MgB_2_–based bulk superconductors and an analysis of the dependence of their properties on technological processes and additions. It is focused on the effects of manufacturing technology parameters, such as pressure, temperature, holding time, impurities, and additions, on the materials’ structure and superconducting characteristics. Below, we present the best-achieved superconducting properties of MgB_2_ bulk materials, such as critical current density and upper critical and irreversibility magnetic fields. Some aspects of the practical application of MgB_2_-based materials are also considered briefly.

## 2. Effect of Manufacturing Pressure–Temperature–Time Conditions on Bulk MgB_2_ Superconducting Characteristics and Structural Features

The superconducting characteristics of MgB_2_ materials depend on many factors and their combination. Very deep and comprehensive studies of the synthesis process of MgB_2_-based materials, the correlation between material structure and superconducting characteristics, and the manufacturing technology have been performed by the authors of [7,8,9,10,11,16,19,73,76,82,84,85,98,99,101,102,103,104,105,106,107,108,109,110,111,112,113,114,115,116,117,118,119,120,121,122,123,124,125,126,127,128,129,130]. These correlations were comprehensively studied for materials prepared using initial powders of MgB_2_ and stoichiometric Mg:2B mixtures (typical characteristics are given in Table 1) at manufacturing temperatures in the range of 600–1100 °C under different pressure conditions using the methods noted above.

To provide the required MgB_2_ stoichiometry, boron powders can be mixed and milled, for example, in a high-speed planetary activator for 3 min with magnesium turnings (noted below as Mg(I)) or magnesium powder < 1 µm (noted below as Mg(II)) [20]. MgB_2_-based materials can be prepared using previously synthesized MgB_2_ powder as well. If a superconducting material is prepared from Mg and B mixtures the process is called synthesis or in-situ, if the material is prepared from MgB_2_ powder it is called sintering or ex-situ.

The critical current density, *J*_c_, of MgB_2_ bulk samples is usually estimated from magnetization measurements using, e.g., a vibrating sample magnetometer (VSM) or a Physical Property Measurement System (PPMS), and the Bean model [102].

The superconducting transition temperature (critical temperature) is estimated using a SQUID magnetometer or four-point method. 

For the VSM measurements on samples with typical sizes of a few mm, the value of *J*_c_ is calculated by using Equation (1):

(1) $$J_{c}=\frac{2∆m}{V\times b_{s}\times (1-b_{s}/3a_{s})}\;,$$

where Δ*m* is the hysteresis of the magnetic moment, *V* is the sample volume, and *a*_s_ and *b*_s_ are the sample dimensions perpendicular to the applied field, with $a_{s}>b_{s}$.

The connectivity, *A*_F_, is estimated from the difference in resistivity at 40 K and 300 K, $\rho_{300}-\rho_{40}$, measured by using the four-point method:

(2) $$A_{F}=\frac{9\mu \Omega \times cm}{\rho_{300}-\rho_{40}}\;,$$

where 9 µΩ·cm is assumed to be the electrical resistivity of MgB_2_ from a polycrystalline sample [6].

The volume pinning force was determined as *J*_c_ *× B* [131].

Below we present the upper critical magnetic, *B*_C2_, and irreversibility, *B*_irr_, fields, which were determined using the four-point method and performing measurements in a 0–15 T field applying a 10–100 mA current [20,85]. The SC shielding fraction can be calculated from the ac susceptibility, with a numerical correction accounting for the demagnetization of the actual sample geometry [109].

**Figure 1:**
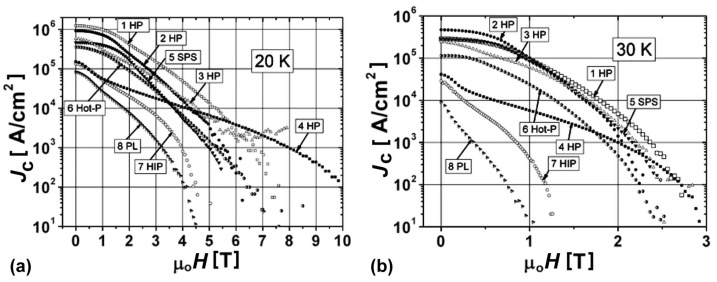
Dependences of critical current density (magnetic measurement), *J*_c_, on magnetic field, μ_o_*H*, for MgB_2_-based materials at 20 K (**a**) and 30 K (**b**) [108]. 1 HP—high-pressure synthesized under 2 GPa at 1050 °C for 1 h from Mg(I):2B(I) with 10% SiC addition; 2 HP—high-pressure synthesized (2 GPa, 1050 °C, 1 h) from Mg(I):2B(I); 3 HP—high-pressure-sintered (2 GPa, 1050 °C, 1 h) from MgB_2_ (VII); 4 HP—high-pressure-synthesized (2 GPa, 600 °C, 1 h) from Mg (II):2B (II); 5 SPS—spark-plasma-synthesized under 50 MPa at 600 °C for 0.3 h and then at 1050 °C for 0.5 h from Mg(I):2B(III); 6 HotP—synthesized by hot pressing (30 MPa, 900 °C, 1 h) from Mg(I):2B(III) with 10% Ta addition; 7 HIP—synthesized under high isostatic (gas) pressure (0.1 GPa, 900 °C, 1 h) from mixture of Mg(I):2B(III) with 10% Ti addition, which was precompacted into a ring shape by broaching; 8 PL—pressureless sintering (in flowing Ar under 0.1 MPa at 800 °C for 2 h) from mixture of Mg(I):2B(III) with 10% Ti addition, which was precompacted into a ring shape by broaching.

The typical dependences of the critical current density, *J*_c_, on an external magnetic field at 20 K and 30 K are presented in Figure 1. Figure 1 presents the highest values found in the literature for bulk MgB_2_-based materials prepared by different methods. These samples were prepared using different initial types of amorphous B and MgB_2_ powders, both without and with the addition of SiC, Ti, and Ta in the amount of 10 wt%, and using boron into which some carbon was specially added during preparation, B(II). The improvement of the critical current density was achieved by the application of a higher manufacturing pressure or a higher pressure of cold compaction (in the case of the following pressureless synthesized samples). The various technologies and initial materials provided the highest critical current density for different ranges of magnetic field and temperature. For example, at 20 K, the sample 1 HP possessed the highest critical current density in relatively low fields, <5 T, it was 4 HP—in a higher field, >5.5 T (Figure 1a). The typical characteristics of MgB_2_-based samples prepared without additions from Mg:2B and MgB_2_ under different conditions were summarized from [98,103,108,119] and are presented in Table 2.

**Figure 2:**
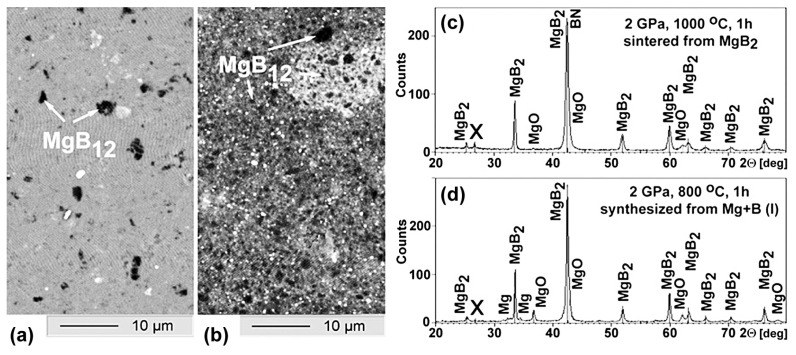
(**a**,**b**)—Sample structures obtained by SEM in COMPO (compositional) contrast: (**a**)—Sample sintered from MgB_2_ (Type VI) under 2 GPa at 1000 °C for 1 h; bright small zones in (**a**) seem to be inclusions (containing O, Zr, Nb, and possibly ZrO_2_) appearing due to milling of initial MgB_2_. (**b**)—Structure of sample synthesized from Mg(I):2B(I) under 2 GPa at 800 °C. (**c**,**d**)—X-ray patterns of these samples, respectively [109].

Figure 2 allows for a comparison of the microstructures of the sintered, ex-situ, and synthesized, in-situ, prepared MgB_2_. One can see that “black” inclusions, which correspond to higher magnesium borides, are present in both materials [109].

The brighter areas on the photos correlate with a higher amount of impurity oxygen, and the darker-looking areas—with a higher concentration of boron in the MgB_2_-based materials.

MgB_12_ inclusions, with sizes up to 10 µm and appearing as the darkest areas in the materials, are randomly distributed. These inclusions are large enough to allow for an estimation of nano-hardness. Using a Berkovich indenter, the nano-hardness of the MgB_2_ matrix and inclusions with stoichiometry near MgB_12_ were studied [20,109]. The inclusion’s nano-hardness of 32.2 ± 1.7 GPa and Young modulus of 385 ± 14 GPa, estimated under a 10–60 mN load, occurred about twice higher than those of the material matrix.

**Figure 3:**
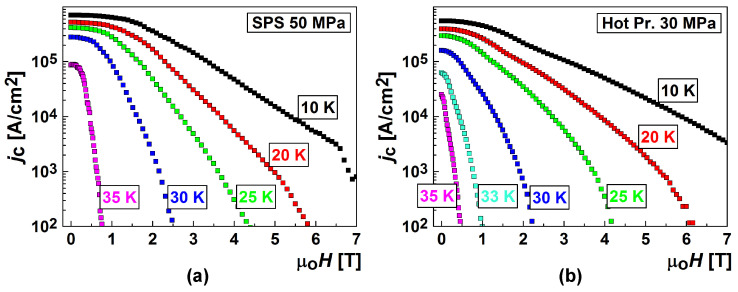
Critical current density, *J*_c,_ vs. magnetic field, μ_o_*H*, of MgB_2_ prepared (**a**) from Mg(I):2B(III) by SPS under 50 MPa at 600 °C for 0.3 h and then at 1050 °C for 0.5 h and (**b**) from Mg(I):2B(III) + 10 wt% Ti by HotP under 30 MPa at 1000 °C for 15 min [119].

**Figure 4:**
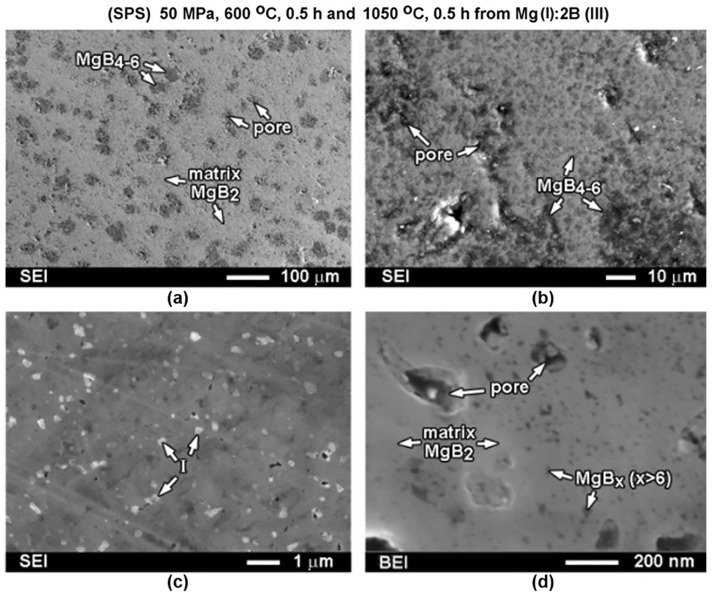
Structures of MgB_2_ materials prepared from Mg(I):2B(III) mixtures under 50 MPa at 600 °C for 0.5 h and then at 1050 °C for 0.5 h. Images were obtained using SEM at different magnifications [109]; (**a**–**c**)—SEI and, (**d**)—BEI.

Figure 3 shows the dependences of the critical current density on a magnetic field at 10–35 K for the samples demonstrating the highest *J*_C_. The samples were prepared from boron of Type III by SPS under an optimal pressure of 50 MPa and HotP under 30 MPa. The highest critical current densities in low magnetic fields were attained in the SPS materials prepared under 50 MPa pressure at 1050 °C, and in the HotP materials—under 30 MPa at 1000–1100 °C [20,119]. The Materials sintered at 1050 °C by the SPS method from preliminarily prepared MgB_2_ powder (Type VII) or ex-situ demonstrated high critical current densities as well, but they were somewhat lower than those prepared from Mg:2B or in-situ (Table 2). The connectivity between the superconducting grains, *A*_F_, and shielding fraction, *S*, (Table 2) were as follows: *A*_F_ = 80% and *S* = 100% for the ex-situ and *A*_F_ = 98% and *S* = 91% for the in-situ SPS prepared materials at 50 MP (at 600 °C for 0.3 h and then at 1050 °C for 0.5 h). The critical current density increased with the synthesis temperature. The explanation for this could be as follow. The material SPS synthesized from Mg(II):2B(III) at 800 °C demonstrates a low density (74% of the theoretical one) and *J*_c_ = 0.4–0.36 MA/cm^2^ in a 0–1 T field at 20 K (Table 2). The density of the material synthesized by SPS from Mg(II):2B(III) at 1050 °C was 94% of the theoretical value, and *J*_c_ = 0.5–0.45 MA/cm^2^ in a 0–1 T field at 20 K. The typical structure of the SPS material is shown in Figure 4. One can observe big porous areas of MgB_4–6_ (Figure 4a,b). Note for all the images: the darkest spots match MgB_x_ (x > 6) inclusions, the matrix with near-MgB_2_ stoichiometry appears as gray; the brightest spots in the figures are Mg-B-O nano-areas, and the dark-gray areas indicate near-MgB_4–6_ stoichiometry.

**Figure 5:**
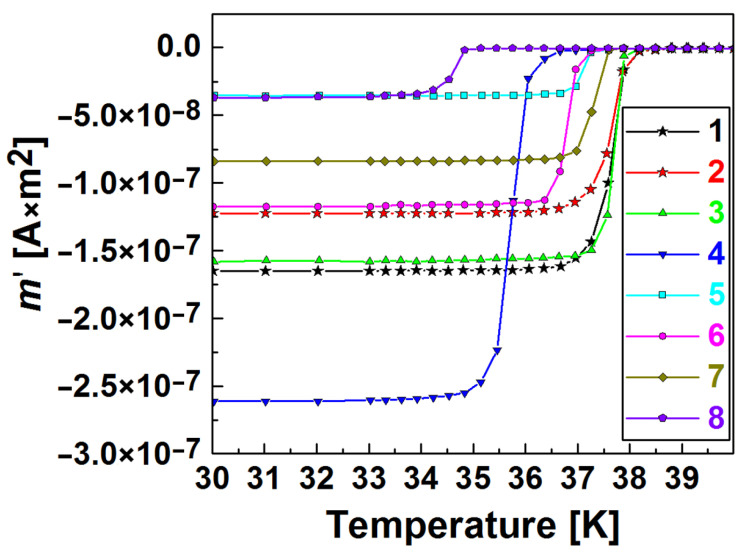
Real (m’) part of the ac susceptibility (magnetic moment) vs. temperature, *T* [108]. Small samples for the study were cut from superconductors prepared under 2 GPa. 1—edge of block 63 mm in diameter, prepared from Mg(I):2B (I and III) + 2 wt% Ti, at 800 °C; 2—center of the same block; 3—block 63 mm in diameter, Mg(I):2B(III) at 950 °C, 4—tablet 9 mm in diameter, Mg(I):2B(V) + 10 wt% Ti, at 1050 °C; 5—tablet 9 mm in diameter, Mg(I):2B(III) + 10 wt% Ti, at 800 °C; 6—tablet 9 mm in diameter, Mg(I):2B(III) + 10 wt% Ti at 1050 °C; 7—tablet 9 mm in diameter, Mg(I):2B(III) + 10 wt% Ta, at 1050 °C; 8—tablet 9 mm in diameter, Mg(II):2B(II) at 600 °C.

Figure 5 shows the temperature dependences of the real part of the ac susceptibility, for some HP-synthesized materials under 2 GPa for 1 h from Mg:2B. The dependences allow for the determination of the temperature of the superconducting transition, *T*_c_, of the materials [108]. The measurements were carried out in an ac magnetic field with 30 μT amplitude, which varied with a frequency of 33 Hz. The critical temperatures of the tested samples were from 34.5 to 38 K.

**Figure 6:**
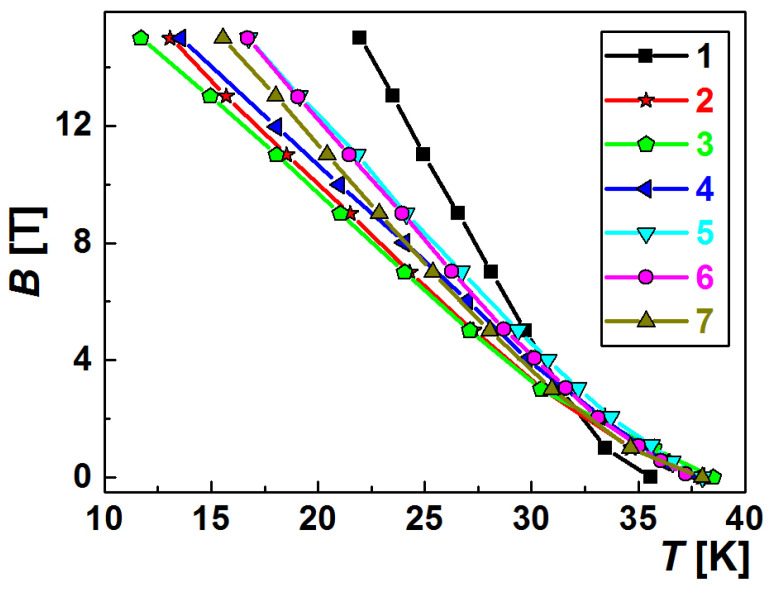
Thermal dependence of the upper critical magnetic field, *B*_c2_, of bulk MgB_2_ [120,132], prepared from: 1—Mg(II):2B(II) under 2 GPa (HP) at 600 °C for 1 h; 2—Mg(I):2B(III) (30 MPa (HotP), 800 °C, 2h); 3—Mg(I):2B(III) (50 MPa (SPS), at 600 °C for 0.3 h and then at 1050 °C for 0.5 h); 4—Mg(I):2B(III) (2 GPa (HP), 900 °C, 1 h); 5—Mg(I):2B(V) + 10 wt% Zr (2 GPa (HP), 800 °C, 1 h); 6—Mg(I):2B(V) + 10 wt% Ti (2 GPa (HP), 800 °C, 1 h); 7—Mg(I):2B(I) + 10 wt% SiC (2 GPa (HP), 1050 °C, 1 h).

Figure 6 presents one of the important characteristics of superconductors, which determines the field of their application, the upper critical magnetic field, *B*_c2_. Figure 6 shows the temperature dependences of the highest upper critical magnetic fields for the HP, SPS, and HotP materials [120,132].

**Figure 7:**
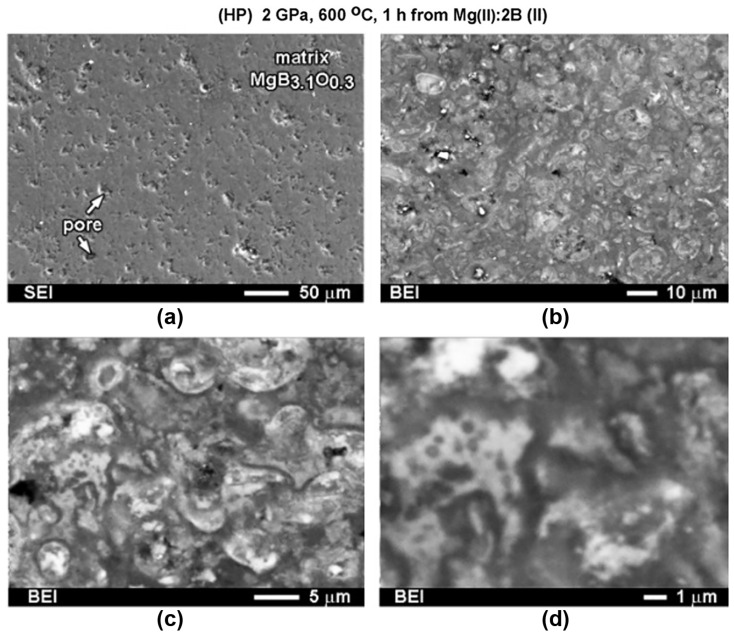
(**a**–**d**)—Microstructures obtained by SEM at different magnifications of MgB_2_ material prepared from Mg(II):2B(II) mixtures under 2 GPa at 600 °C for 1 h [109]. (**a**)—SEI and, (**b**–**d**)—BEI.

Let us consider, as an example, the structure of the sample prepared from Mg(II):2B(II) (boron with C addition) at 600 °C under 2 GPa (Figure 1, curve 4HP). The sample demonstrates a low critical temperature, *T*_c_, of about 34.5 K (Figure 5, curve 8) and possesses a low connectivity, *A*_F_ = 18%, and density (Table 2, line 5). Despite the low noted properties, the sample demonstrates the highest critical current density in a magnetic field range of 6–10 T at 20 K (Figure 1, curve 4 HP), and the highest upper critical magnetic field, *B*_c2,_ of 15 T at 22 K (Figure 6, curve 1) presented in the literature. An extrapolation give a *B*_c2_ of 42 T at 0 K. Figure 7 shows the structure of this material under different magnifications.

### 2.1. Effect of Manufacturing Pressure

**Figure 8:**
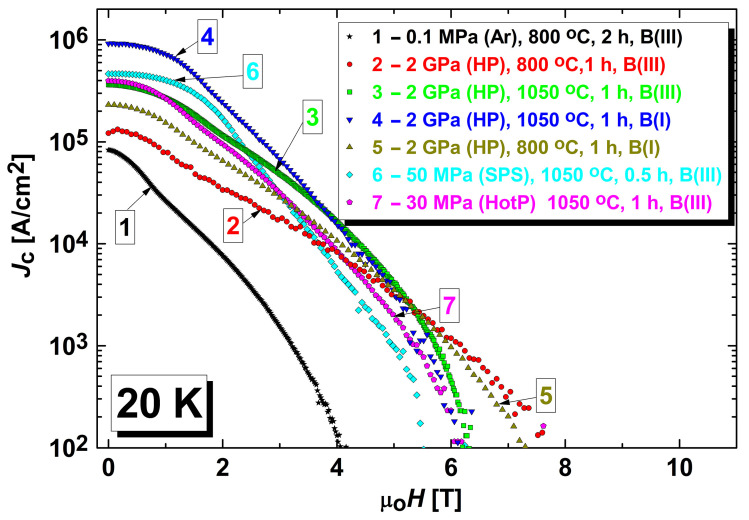
The dependences of critical current density, *J*_c_, at 20 K on a magnetic field. The MgB_2_ samples were prepared from Mg(I):2B(I) and Mg(I):2B(III). The graph was composed using the data presented in [20,98,103,119].

Usually, a higher manufacturing pressure allows to achieve a higher critical current density for materials both without and with additions due to an increase in the materialʹs density and connectivity between superconducting grains (Table 2) [20,98,103,108,109,119]. Figure 8 presents the dependences of critical current density vs. external magnetic field for the MgB_2_-based materials prepared from the same Mg(I):2B(III) mixture by different methods at 800 and 1050 °C, and under different pressures: 0.1 MPa (PL), 2 GPa (HP), 50 MPa (SPS), and 30 MPa (HP). A comparison of curves 1 and 2, as well as of curves 3, 4, 5, and 6, demonstrates the positive effect of a pressure increase.

During synthesis in a flow of Ar at 1050 °C and under a pressure of 0.1 MPa, some amount of Mg evaporated after 15 min of heating at 1050 °C. X-ray diffraction studies have revealed that the matrix of the synthesized material acquires the structure of MgB_4_ [109]. The sample prepared under such conditions was non-superconducting. Previously, it has been shown that cold densification at 2 GPa does not improve results. However, high-pressure-synthesized materials under 2 GPa at 800 and 1050 °C have MgB_2_ matrices and demonstrate high critical currents. After a 15 min holding time at 1050 °C in flowing Ar under 0.1 MPa, some amount of Mg evaporates and non-superconducting MgB_4_ is formed (instead of MgB_2_). An increase in the holding time of up to 2 h at 1050 °C results in more intensive Mg evaporation and formation of the MgB_7_ matrix phase, which is non-superconducting as well [109].

In the materials synthesized in flowing Ar under 0.1 MPa, using SPS under 50 MPa, and HP under 2 GPa, one can observe grains of higher magnesium borides MgBx (x = 4–20), which look the blackest in photos of the microstructures. MgB_x_ (x = 4–20) phase inclusions are larger, and their amount is higher in materials produced at low temperatures compared to materials produced at high temperatures.

### 2.2. Effect of Manufacturing Temperature

**Figure 9:**
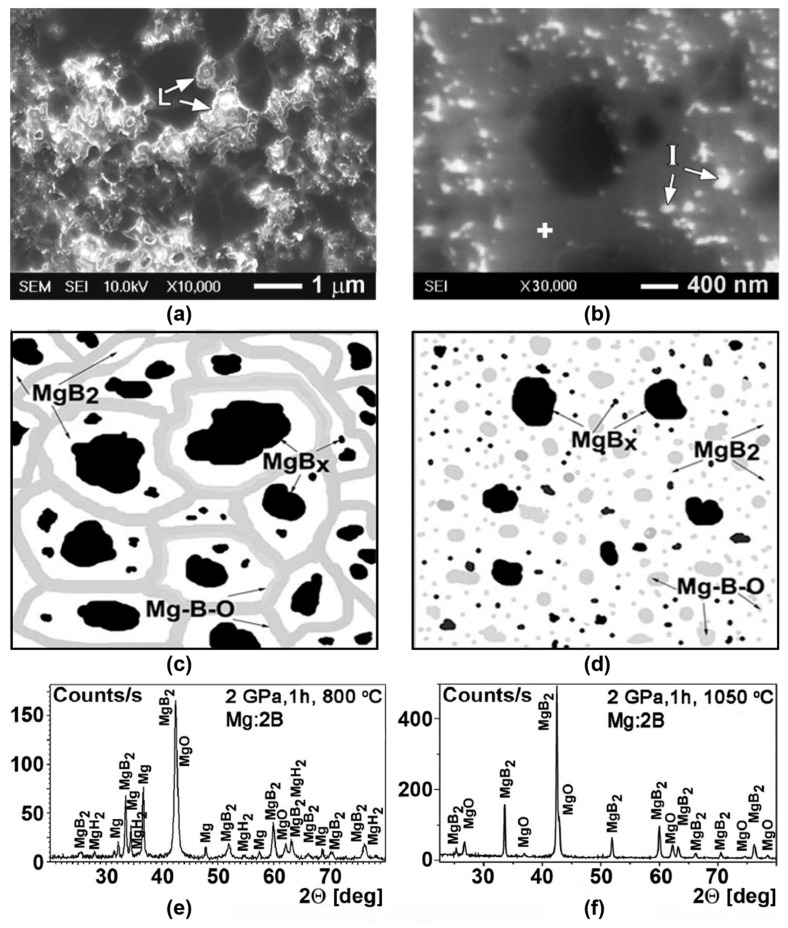
(**a**,**b**)—SEM images in SEI mode of MgB_2_ materials synthesized from Mg(I):2B(III) mixtures under 2 GPa, for 1 h at 800 and 1050 °C, respectively [109]. (**c**,**d**)—Schema of MgB_2_-based material structures synthesized at low temperature of 800 °C (**e**) and high temperature of 1050 °C (**f**) [85]. (**e**,**f**)—X-ray patterns of samples shown in (**a**,**b**) [113].

One important factor influencing the superconducting properties of MgB_2_ bulk material is the manufacturing temperature. The dependences of the superconducting properties on the manufacturing temperature are associated with variations in the MgB_2_ structures [57,58,59,60,61,62,63,64,65,66,67,68,69,70,71,72,73,74,75,76,77,78,79,80,81,85,109,110,117]. The typical structures of MgB_2_ materials synthesized at low (800 °C) and high (1050 °C) temperatures under 2 GPa are shown in Figure 9a,b [132].

The X-ray analysis of both MgB_2_-based materials shows that they contain MgB_2_ and MgO phases. However, SEM and EDX analyses and an Auger spectroscopy study indicate the presence of three main phases in the materials: (1) a matrix with near–MgB_2_ stoichiometry, which contains a small amount of an impurity of oxygen (grey areas in the photo, Figure 9a,b); (2) inclusions (grains) of higher magnesium borides, MgB_x_, x >> 2, look the blackest; and (3) oxygen–enriched places look brightest or white, indicating Mg-B-O inclusions.

The forms of the Mg-B-O inclusions depend on the manufacturing temperature and are principally different. In the MgB_2_ material synthesized at low (800 °C) temperature, their forms are nanolayers noted by “L” in Figure 9a, and at high (1050 °C) temperature they are separate inclusions, noted by “I” in Figure 9b [109]. The difference is schematically shown in Figure 9c,d. The MgBO inclusions can play the role of pinning centers and the difference in their structures is reflected in the different dependencies of the critical current densities on the magnetic field. Moreover, the effect of oxygen aggregation with the manufacturing temperature increases. Besides, the reduction with temperature in the amount and sizes of higher magnesium borides inclusions (which appear the most black) has been observed. 

The manufacturing temperature of MgB_2_ superconductors can be varied in a rather wide temperature range of 600–1200 °C. The application of a higher pressure allows for an increase in the manufacturing temperature of MgB_2_ superconductors because higher pressures prevent the evaporation of magnesium at higher temperatures, and the following changes in the materialʹs stoichiometry.

**Figure 10:**
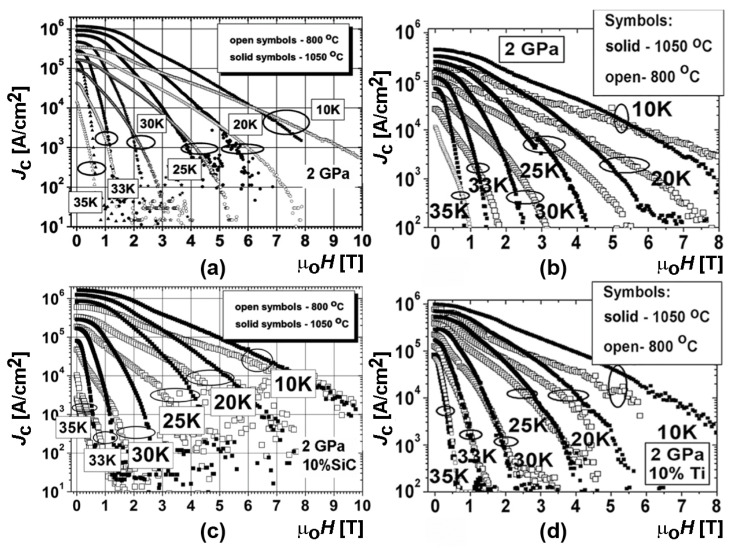
Critical current density, *J*_c_, vs. magnetic field, µ_o_*H*, of MgB_2_ materials prepared from Mg(I):2B(I) and Mg(I):2B(III) mixtures under 2 GPa, at 800 and 1050 °C for 1 h (**a**,**b**), respectively; additions of SiC (0.2–0.8 μm) to Mg(I):2B(I) mixture (**c**) and Ti (99%, 1–3 μm) to Mg(I):2B(III) (**d**) [103].

As example of the manufacturing temperature influence, Figure 10 presents the critical current densities of the materials synthesized from different types of initial boron without and with Ti and SiC additions at the low (800 °C) and high (1050 °C) temperatures. One can see that the synthesis at the low temperature allows for the achievement of higher critical currents in higher magnetic fields. However, the synthesis at the high temperature leads to higher critical currents in low magnetic fields. This is observed for a temperature range from 10 to 35 K and in external magnetic fields up to 10 T [20,103,109].

### 2.3. Pressure—Temperature Effect on Pinning in MgB_2_

**Figure 11:**
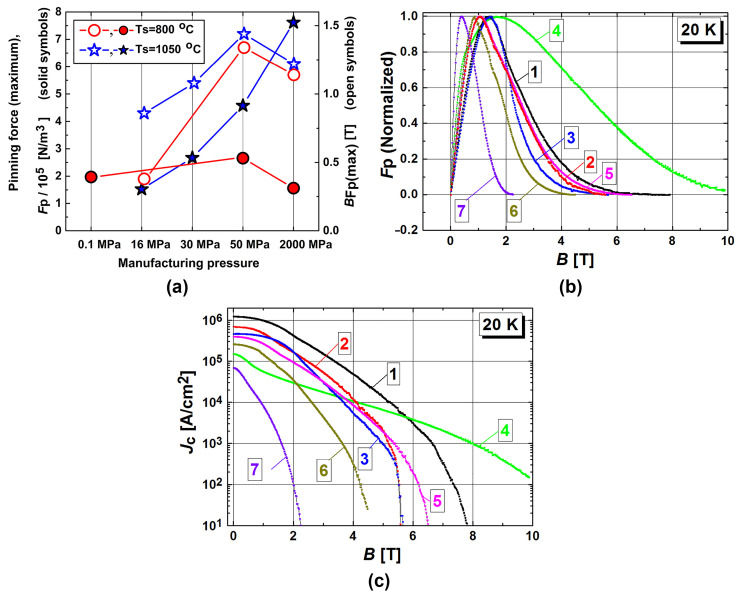
(**a**) Maximal pinning forces, BFp(max), and corresponding values of magnetic fields at 20 K vs. synthesis pressure for MgB_2_-based materials synthesized from Mg(I) and B(III) at 800 (circles) and 1050 °C (stars); (**b**)—normalized pinning force, *F*_p_, vs. magnetic field, *B*, calculated from the critical current density, *J*_c_; and (**c**)—dependence of critical current density, *J*_c_, on magnetic field. Designations: *k* = B_peak_/B_n_; PP—point pinning; GBP—grain boundary pinning; and MP—mixed pinning [128]. Curves: (1) Mg(I):2B(I) + 10% SiC, 2GPa, 1050 °C, 1 h, k = 0.51 (PP); (2) Mg(I):2B(III) + 10% Ti, 2 GPa, 1050 °C, 1 h, k = 0.42 (MP); (3) Mg(I):2B(III), 50 MPa, 600 °C for 0.3 h and then 1050 °C for 0.5 h, k = 0.63 (>PP?); (4) Mg(II):2B(II) with 3.5% C, 2 GPa, 600 °C, 1 h, k = 0.31 (GBP); (5) Mg(I):2B(III) + 10% Ti, 30 MPa for 1 h and then 1000 °C for 0.2 h, k = 0.42 (MP); (6) MgB_2_, 16 MPa, 1150 °C, 0.3 h, k = 0.45 (PP); (7) Mg(I):2B(III), in flowing Ar atmosphere under 0.1 MPa, 800 °C, 4 h, k = 0.35 (GBP).

The pinning force was estimated and the types of dominant pinning were determined for the MgB_2_–based superconductors in [7,69,71,91,128,131]. Table 3 and Figure 11 summarize the results of these studies, which were presented in [7,128]. The materials tested in these works were prepared under different pressure—temperature conditions. The dominant pinning mechanism was determined using the method proposed in [131]. This mechanism was determined using the volume pinning force *J*_c_ *× B*, according to the following procedure: “The field *B*_peak_, where the maximum of the volume pinning force *F*_p_ takes place, is normalized by the field *B*_n_, at which the volume pinning force drops to half its maximum (on the high external field side). The position of the peak, *k* = *B*_peak_/*B*_n_, is expected to be at 0.34 and 0.47 for grain boundary pinning (GBP) and point pinning (PP), respectively”.

Figure 11a shows the typical dependences of the maximal pinning force and field, *B*_n_, at 20 K on the manufacturing pressure and temperature. At the low temperature (800 °C), there is the maximum volume pinning force at a manufacturing pressure of 50 MPa. At the high temperature (1050 °C), this force increases monotonically with the pressure [128]. An increase in pressure (up to 2 GPa) usually leads to a reduction in porosity (from 47% to 1%) and, as noted above, to an enhancement of the critical current density. *F*_p_(max) is also increased by the addition of Ti or SiC, both in the low- and the high-temperature synthesized materials (Table 3). The pinning forces in the in-situ prepared samples are higher than those in the ex-situ ones. The position of *F*_p_(max) shifts to higher magnetic fields with the manufacturing pressure and due to the addition of Ti or SiC. A shift has also been observed in the case of using the in-situ preparation (compared to the ex-situ) [98]. The pinning type GBP dominates in the materials prepared at low temperatures (600–800 °C), while the high-temperature preparation results mainly in PP or intermediate behavior, so-called mixed pinning (MP). Exceptions have been found for materials produced by SPS (the *k* values were too high for the PP mechanism). These materials contain a wide range of higher magnesium borides, MgB_x_ (x = 4–20), within their structure [20,109,119,128].

The studies of the samples prepared under a pressure in the range of 16–96 MPa have showed that a manufacturing pressure of about 50 MPa turns out to be optimal for the SPS synthesis method.

The samples with different magnetic fields, *B*_peak_, corresponding to the maximum pinning force, *F*_p_ demonstrate different behaviors of the critical current density. An increase in the magnetic field, *B*_peak_, usually leads to a decrease in the critical currents in low fields, and a significantly slower reduction with an increasing field (compare, e.g., curves 1 and 4 in Figure 11b,c).

## 3. Characteristics of Initial Compounds and Critical Current Densities

The grain boundaries and the amount of impurity oxygen can influence the pinning and critical current density of the synthesized (in-situ) and sintered (ex-situ) magnesium diboride-based materials [103].

In previous publications, the following correlations have been assumed to be important for changing the superconducting characteristics of materials based on magnesium diboride:

- the amount of oxygen in the initial boron and magnesium diboride powders and the oxygen concentration in the superconducting matrices of MgB_2_ bulk materials;
- the average grain sizes of the initial boron and magnesium diboride and the average sizes of the grains in the superconducting phase;
- the amount of oxygen in and the grain sizes of the initial components and the critical current density;
- the oxygen amount and the grain sizes in the prepared superconducting materials and the critical current densities.

The authors of [103] demonstrated that no correlation could be found between the average grain size (in the range of 0.8–9 µm) and the impurity oxygen content (0.66–1.9 wt%) in the different initial B or MgB_2_ powders and the amount of oxygen in the superconducting bulk MgB_2_ prepared using HP. The oxygen content (estimated by SEM EDX) in the in situ prepared MgB_2_ was 7–24 wt% and in the ex-situ it was 4–12 wt%.

The grain boundaries in MgB_2_ can be considered as pinning centers for Abrikosov vortices. The higher density of the pinning centers leads to a higher critical current density, *J*_c_. Smaller grains and, thus, a higher total surface of grain boundaries in MgB_2_ should provide stronger pinning and a higher *J*_c_.

The critical current density and, average crystal sizes, calculated from the line broadening of the MgB_2_ phase in the X-ray diffraction patterns (Equation (3)) and lattice parameters of the MgB_2_ phase for ex-situ and in-situ prepared materials under 2 GPa are presented in Table 4.

The average crystallite sizes of bulk MgB_2_-based superconductors were calculated from the line broadening of the MgB_2_ phase in the X-ray diffraction patterns by the standard program as follows:

(3) $$\begin{array}{cc} & Crystallite\;size=\frac{K\times \lambda }{W_{\mathrm{size}}\times \cos\theta },\\ \mathrm{with} & \\ & W_{\mathrm{size}}=W_{b}-W_{s}\;, \end{array}$$

where: *W*_size_—the broadening caused by small crystallites; *W*_b_—broadened profile width; *W*_s_—standard profile width of 0.08°; *K*—shape factor; λ—X-ray wavelength. The value of the K factor in Scherrer’s equation was set by default to 0.9 [103].

There were no correlations between the sizes of the crystallites (grains) in manufactured bulk MgB_2_ and the critical current density, *J*_c_ (at 10 and 20 K in a 1T field), for both the in-situ and ex-situ superconductors manufactured under a pressure of 2 GPa (Table 4) [20,103].

The average crystallite (or grain) sizes of MgB_2_ obtained using the HP method increased slightly with the preparation temperature (for example, in the range of 700–1000 °C, Table 4), especially for MgB_2_ obtained in-situ (from 15 to 37 nm), and less for that obtained ex-situ (from 18.5 to 25 nm) [103]. The in-situ MgB_2_ with somewhat bigger crystallites demonstrated a higher *J*_c_ that looks contradictive. The explanation may be that *J*_c_ may be influenced in parallel by other factors. The critical current density can also be strongly influenced by the distribution of impurity oxygen in the MgB_2_ structure and the formation of inclusions of higher magnesium borides, which are also affected by the production temperature. This is discussed in this review below.

Up to now, it has not been entirely clear which set of characteristics, of the initial boron or MgB_2_, could give a guarantee for achieving a high critical current density in bulk MgB_2_ superconductors. Of course, the high level of their purity is very important, but does not give a hundred–percent guarantee of high quality from the point of view of the superconductive characteristics of the synthesized superconductors.

### Effect of Mg:xB (x = 4–20) Ratio of Powdered Mixture on Microstructure and Characteristics of HP-Synthesized Materials

The authors of [103,108,125] have studied the efect of the boron concentration in the initial mixtures on the structure and superconducting properties of the HP-synthesized materials.

The concentration of boron in the MgB_x_ inclusions, which are present in the MgB_2_ matrix, varies in a wide range. Along with the superconducting MgB_2_, there exist several stable, non-superconducting, higher magnesium borides (MgB_4_, MgB_7_, MgB_12_, MgB_17_, MgB_20_, and Mg_2_B_25_). The higher magnesium borides can crystallize in the MgB_2_ matrix and can affect pinning. By changing the pressure—temperature—time conditions, one can change the stoichiometry of the higher borides inclusions and the areas they occupy in the MgB_2_ matrix. Higher magnesium borides MgB_x_ in the high–pressure (2 GPa) manufactured materials demonstrate x = 9–14, and mostly around 12. In spark plasma manufactured materials, the MgB_x_ phases with x = 4–6 occupy rather porous and rather large areas, which appear as the gray areas in Figure 4a,b. Small inclusions, with x = 8–16, are also present in the material and are shown as the black areas in Figure 4. The MgB_x_ inclusions with x= 6–8 are in the materials synthesized by the hot–pressing method. This allows for the assumption that pressure plays an essential role in the stoichiometry of MgBx inclusions of high magnesium borides. The inclusions with x = 18–25, or even pure B, appear in the structure randomly and, thus, cannot influence the material characteristics as a whole [109].

MgB_x_ inclusions are practically “invisible” to a traditional X-ray diffraction analysis despite the essentially different amounts of boron, the crystallographic structures of higher magnesium borides, and their properties (e.g., nano-hardness). The reason could be due to their fine dispersion in the material structure and the large number of atoms in unit cells of low symmetry, which results in a high amount of “reflecting planes”. This essentially reduces the intensities of the X-ray reflections from higher magnesium boride grains randomly distributed in the MgB_2_ matrix, which cannot be seen on the background of the very strong reflections from MgB_2_ [109].

**Figure 12:**
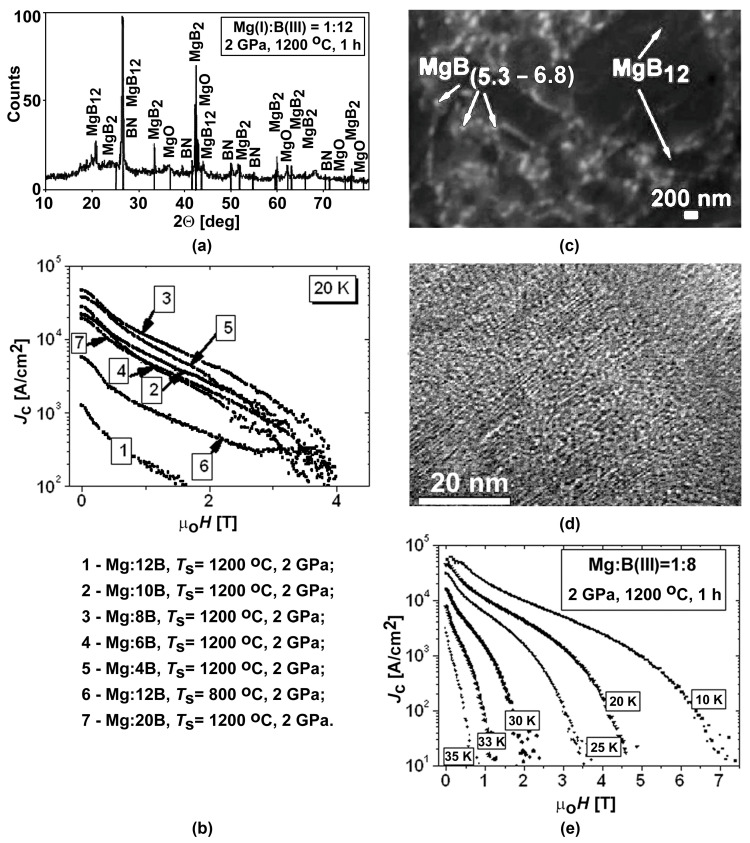
(**a**)—X-ray patterns of the material synthesized under 2 GPa at 1200 °C for 1 h from Mg(I):12B(III); (**b**)—dependences of *J*_c_ on the external magnetic fields, μ_o_*H*, at 20 K for the materials synthesized under 2 GPa for 1 h from Mg(I) and B(III), taken in the ratio Mg:xB, and synthesized at temperature, *T*_S_: curves 1—Mg:12B, *T*_S_ = 1200 °C; curve 2—Mg:10B, *T*_S_ = 1200 °C; curve 3—Mg:8B, *T*_S_ = 1200 °C; curve 4—Mg:6B, *T*_S_ = 1200 °C; curve 5—Mg:4B, *T*_S_ = 1200 °C; curve 6—Mg:12B, *T*_S_ = 800 °C; curve 7—Mg:20B, *T*_S_ = 1200 °C; (**c**)—backscattering SEM image of the material prepared under 2 GPa at 1200 °C for 1 h from Mg(I):12B(III); (**d**) HRT—EM microstructure (of a MgB_12_ grain, the stoichiometry of which was estimated by HRTEM EDX); (**e**)—dependences of critical current density, *J*_c_, on magnetic fields, μ_o_*H*, at 10–35 K for the materials prepared under 2 GPa at 1200 °C for 1 h from mixtures of Mg(I):8B(III) [103].

The study of the influence of boron concentration on the superconducting material properties has been performed using initial mixtures of Mg (I) and B(III) [103,108,125]. The components were mixed and milled in a high-speed planetary activator for 3 min with steel balls, and then the materials were synthesized under 2 GPa at 800 and 1050 °C for 1 h. The following mixtures were investigated: Mg(I):4B(III), Mg(I):6B(III), Mg(I):8B(III), Mg(I):10B(III), Mg(I):12B(III), and Mg(I):20B(III). The results for the critical current, *J*_c_, and temperature, *T*_c_, obtained by a vibrating sample magnetometer and PPMS are shown in Figure 12. Rather high critical current densities (Figure 12c,d), as well as a transition superconducting temperature of about 35 K (Figure 12b), were estimated from magnetization loops of the materials prepared from Mg(I) and B(II) mixtures, taken in Mg:8B and even Mg:20B proportions. For example, an X-ray analysis showed that a high amount of the MgB_2_ phase was present in materials prepared from the Mg:8B (Figure 12b,e) and Mg:12B (Figure 12a–c) mixtures. However, the study using the four-probe method allowed for the conclusion that there was no transport current flowing through the samples [103,108,125]. Figure 12d demonstrates the microstructure obtained by an TEM of a MgB_12_ grain, the stoichiometry of which was estimated by TEM EDX. 

## 4. Effect of Additions on Structure and Superconductive Characteristics of MgB_2_

As mentioned in Introduction, for more than 20 years, since the discovery of the superconductivity in MgB_2_, scientists have been exploring the possibility of increasing the pinning and, hence, the critical current density using various additives [38,39,40,41,42,43,44,45,46,47,48,49,50,51,52,53,54,55,56,57,58,59,60,61,62,63,64,65,66,67,68,69,70,71,72,73,74,75,76,77,78,79,80,81,82,83,84,85,86,87,88,89,90,91,92,93,94,95,96,97,98,99,100]. The positive effects of C, C-containing compounds, Ti, Ta, Zr, compounds (borides and carbides) containing these metals, SiC, BN, Si_3_N_4_, NbB_2_, Dy_2_O_3_, SnO_2_, Sn-O, Ti-O, Rb_2_CO_3_, Cs_2_CO_3_, etc., have been reported. However, the discovered effects of some additives, such as SnO_2_, Sn-O, and Dy_2_O_3_ [94,95,96,97,98,121], have appeared contradictory due to a combination of factors acting in parallel. In some cases, a significant improvement has been achieved by increasing the density of materials without additives, or their effect has been negligible and lies within the range of measurement error. The authors of [94,95,96,97] have claimed that additions of SnO_2_ and Dy_2_O_3_ can lead to critical current density increase, but the authors of [98,121] have demonstrated that these oxygen–containing additions reduce the critical current density or do not lead to its notable change. Here, we give a more detailed description of the effects of C, Ti, TiH_2_, Ta, Zr, SiC, and Ti-O, since in our opinion their effects have received more confirmations in the literature.

### 4.1. Effect of Ti, Ta, Zr, and TiH_2_ Additions

In earlier publications [81,133,134], the positive effect of Ti and Zr additions on the critical current density has been explained by the formation of TiB_2_ and ZrB_2_ inclusions into thin (atomic-size) layers, which improve pinning. However, the mechanism of the Ti and Zr additions influence has not been proven experimentally. The positive effect of SiC additions has been explained in [69,70,71,72,74] by the following: the carbon in the MgB_2_ structure is solved after the decomposition of SiC into C and Si, the latter forming Mg_2_Si. The SiC additive acts as a source of carbon. Carbon, in small amounts, can form a solid solution in the superconducting MgB_2_ phase, somewhat decreasing the transition temperature, but essentially increasing the upper critical magnetic and irreversible fields, i.e., increasing the critical current density in high magnetic fields.

**Figure 13:**
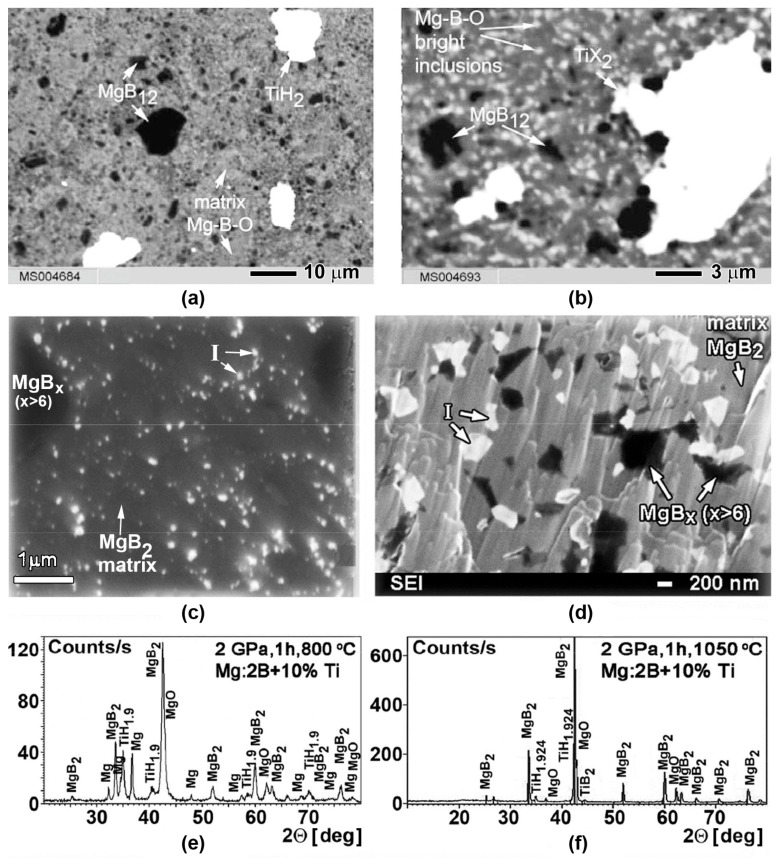
Microstructures of the materials synthesized from Mg(I):B(III) with a 10 wt% of Ti (3–10 μm) addition under 2 GPa for 1 h at 800 (**a**,**c**) and 1050 °C (**b**,**d**) [108]. X-ray patterns of these materials (**e**,**f**). (**c**,**d**) show the places where Ti is absent [103,113].

The review of publications [84,103,108,110,111,125,135,136,137,138], in which the influence of Ti, Zr, and Ta additions are studied, have shown that the effects of these additions are different from that of SiC additions. No diffusion of Ti, Ta, or Zr into the MgB_2_ was found in the samples prepared under 2–3 GPa at 700–1100 °C [83,125] and, as a result, the inclusions of phases containing Ti or Ta are rather too big and randomly distributed to be efficient pinning centers (Figure 13a,b). However, the presence of Ti or Ta causes an increase in the amount of inclusions with a stoichiometry near that of MgB_x_ (x~12) in HP-prepared materials (Table 5) [53,55,95]. At low synthesis temperatures (700–850 °C under 2 GPa), Ta and Ti transform into hydrides due to adsorbing impurity hydrogen (Figure 13e), which may come from the atmosphere or fro materials which were in contact with the Mg-B mixture during mixing or synthesis. Therefore, these additions prevent the formation of MgH_2_ (Figure 9f), the presence of which decreases the critical current [53,55]. The X-ray diffraction patterns shown in Figure 13e,f indicate that when Ti is added to a mixture of magnesium and boron, TiH_2_ is formed along with magnesium diboride and an admixture of magnesium oxide. MgH_2_ is not formed. The formation of only one titanium-containing phase, TiH_1,924_, in the materials prepared under 2 GPa at 800 °C has been confirmed by TEM and NanoSIMS ion mapping [139]. This fact looks unusual from the point of view of thermodynamics, because the titanium hydride (TiH_2_) formation enthalpy of −15.0 kJ mol^−1^ is higher than that of the formation of titanium boride (TiB_2_: from −150 to −314 kJ mol^−1^) or oxides (TiO_2_: −944.057 kJ mol^−1^; Ti_2_O_3_: −1520.9 kJ mol^−1^; and Ti_3_O_5_: −2459.4 kJ mol^−1^) [123]. There is a lot of impurity oxygen in the material and it contains boron, but only TiH_2_ is formed at a low synthesis temperature [139]. At higher synthesis temperatures, TiH_1,924_ and TiB_2_ form (Figure 13f).

**Figure 14:**
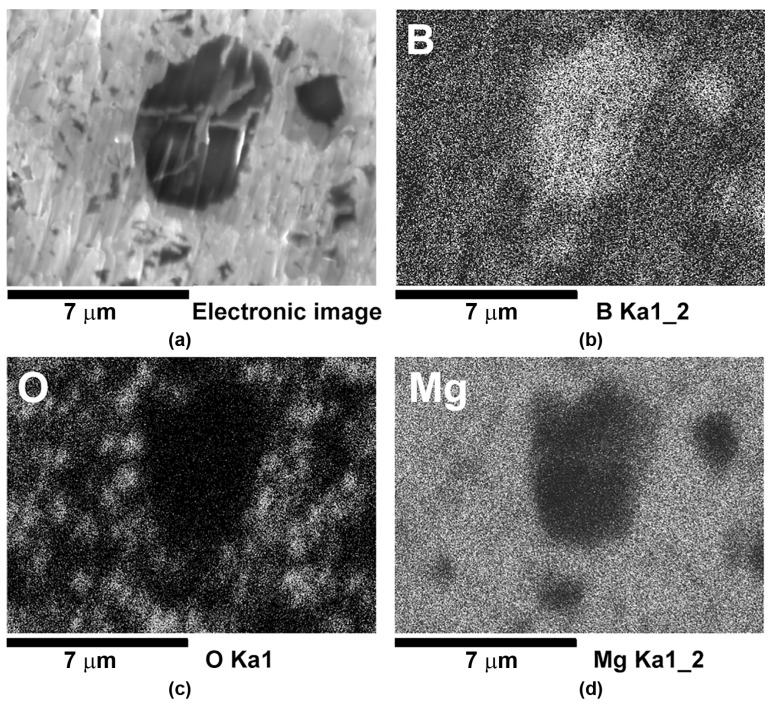
(**a**) Image of microstructure of MgB_2_ sample with 10 wt% of Ti (3–10 μm); image 16a was taken in the place where the Ti grains are absent. (**b**–**d**)—EDX maps of boron, oxygen, and magnesium distributions over the area of the image shown in 16e (the brighter the area looks, the higher the amount of the element under study) [103].

The typical distribution of Mg, B, and O in the structure of MgB_2_-based materials prepared from Mg(I):2 B(III) with 10 wt% of Ti (3–10 μm), in the phase where Ti grains are absent, is shown in Figure 14.

The absorption of hydrogen and, thus, the prevention of the formation of MgH_2_ by Ta and Zr additions, has been observed, as in the case of Ti additions [103]. However, Ti is the most powerful absorbent of these three metals. Note also that the additions of Ti to the MgB_2_ mixture, or even the synthesis of a big MgB_2_ block wrapped in a Ti foil, prevents an MgB_2_ sample from cracking due to the absorption of impurity hydrogen by Ti.

**Figure 15:**
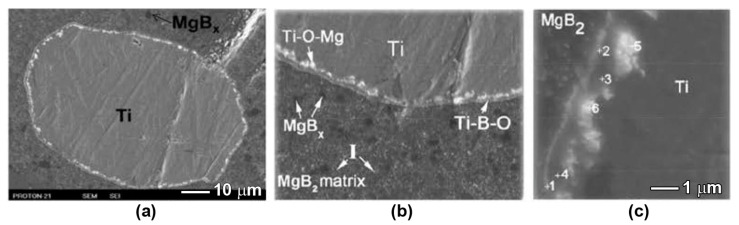
(**a**–**c**) SEM images of MgB_2_ sample with 10 wt% of Ti powder (about 60 μm) synthesized under 2 GPa at 800 °C for 1 h: SEI (**a**–**c**) [113]. Notations: “I”—Mg-B-O inclusions, MgB_x_—higher magnesium borides. In (**c**), the points marked by No. 1–6 are the points for which were made quantitative Auger analyses, the results of which are summarized in Table 6 [113].

When Ti and Ta were added to the initial Mg:2B mixture, in addition to hydrogen absorption, another effect was also observed. Additions of Ti and Ta promote the formation of higher magnesium boride inclusions [103]. Within the structure of MgB_2_ materials synthesized using the HP method with Ti and Ta additives (Table 5), a larger amount (*N*) of the magnesium boride phase with a stoichiometry close to MgB_12_ was observed, compared to the material without additives. A higher amount of the higher magnesium boride phase correlates with higher critical currents in the 1 T field. So, the addition of Ti can affect the boron distribution in MgB_2_-based material. This can be seen in Figure 15b, for example, where around the Ti inclusions the density of the black inclusions (higher magnesium borides) is much higher.

At a low synthesis temperature (800 °C), in MgB_2_-based materials synthesized using the HP method, Ti promotes the aggregation of oxygen into individual oxygen-enriched Mg-B-O inclusions, in contrast to the material without additives containing Mg-B-O nanolayers (Figure 13c) The average amount of oxygen is about 5 wt% in the matrix of the sample with Ti addition (as SEM EDX showed), while in the matrix of the material without Ti additions and with Mg-B-O nanolayers, it is about 8 wt%.

Although, there is not yet a complete understanding of the mechanism of the influence of titanium on the characteristics of MgB_2_, a material based on MgB_2_ with titanium additives with large (about 60 μm, Figure 15) grains has provided some insight into the processes occurring during synthesis. An analysis of the interaction zones around the titanium grains (Figure 15, Table 6) allows us to come closer to an explanation of the observed oxygen and boron redistributions caused by Ti addition. As mentioned above, the density of the location of higher magnesium boride inclusions, MgB_x_, is higher around Ti grains than in the MgB_2_ matrix (Figure 15b). The inclusions (which look brightest in Figure 15), enriched by magnesium and oxygen, are observed inside the Ti grain near its boundary, which were formed as a result of Mg and O diffusion. The Mg-B-O inclusions with a somewhat smaller amount of oxygen (points 1, 2 in Figure 15c) than in the inclusions (points 5, 6 in Figure 15c) are observed near the grain boundary, inside of the Ti-containing grain. Magnesium defuses into titanium more intensively than boron (compare points 3, 4 and points 5, 6 in Figure 15c and Table 6) [113]. Magnesium and oxygen diffuse deeper into the Ti grain (Figure 15) than boron, and this could be an explanation for the redistributions of boron and oxygen in MgB_2_, and possibly the reason for the higher magnesium boride grains formation. A layer containing boron is located the nearest to the boundaries inside the Ti grain (points 3 and 4 in Figure 15c and Table 6).

To summarize the influence of Ti addition on the structure and characteristics of the MgB_2_-based materials, we conclude the following. (1) The impurity of hydrogen is adsorbed by Ti. (2) A redistribution of the impurity of oxygen is caused, i.e., the effect of the titanium additive is similar to that of an increase in preparation temperature. Note that if titanium is added, oxygen aggregation occurs even at a low synthesis temperature. (3) Ti addition increases the number of inclusions of higher magnesium borides, MgB_x_ (x > 4).

**Figure 16:**
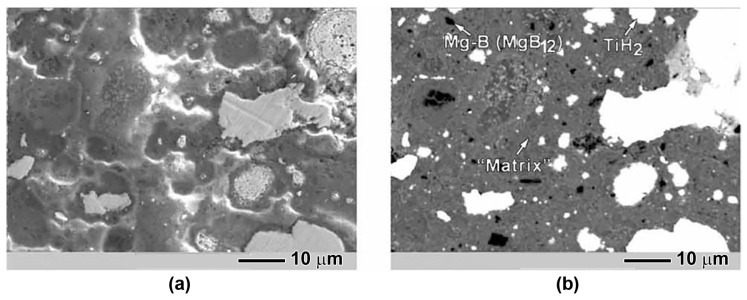
(**a**,**b**)—Microstructure of magnesium diboride synthesized from Mg(I):B(III) with 10 wt% TiH_2_ addition under 2 GPa at 950 °C for 1 h in SEI [84] (**a**) and COMPO (**b**) regimes.

The TiH_2_ phase is present in both the low- and the high-temperature-synthesized materials as detected by X-ray diffraction. TiH_2_ coexists along with TiB_2_ in the high-temperature-synthesized samples. In the case where TiH_2_, in the amount of 10 wt%, was specially added to the Mg:2B mixture [84], a high porosity after synthesis (Figure 16a) was observed. The high porosity results in an essential reduction (by more than two orders) in the critical current density in comparison to the materials without this addition.

### 4.2. Effect of SiC Additions

**Figure 17:**
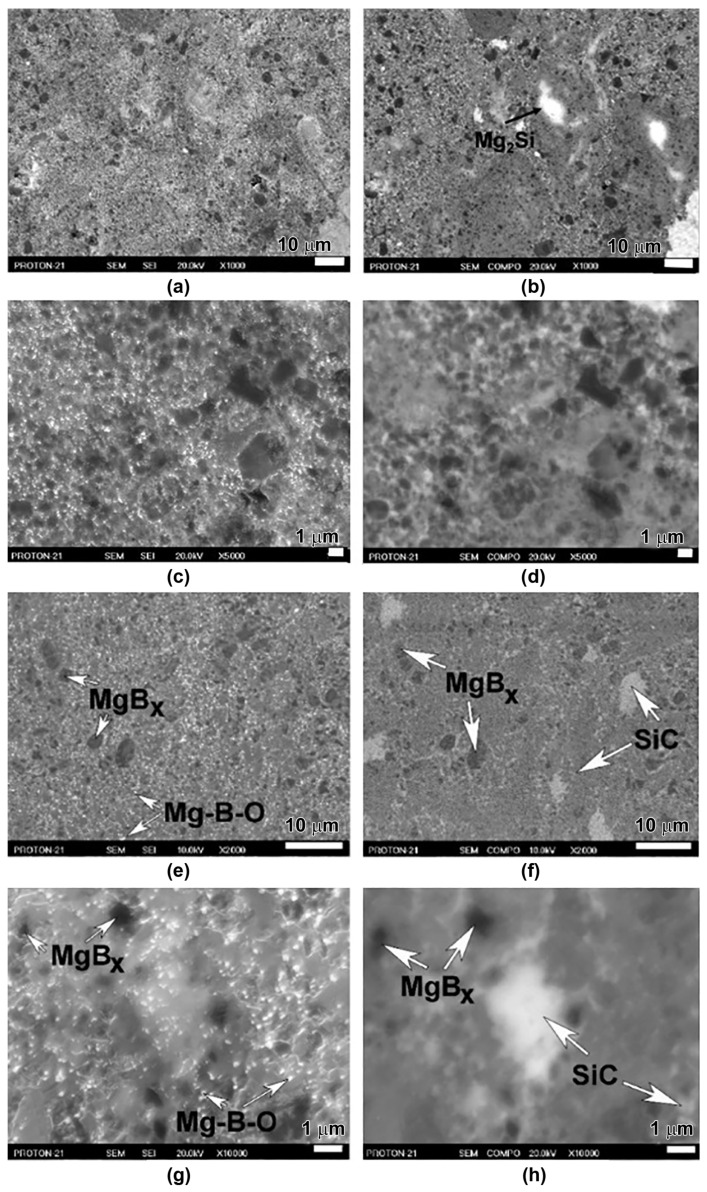
Microstructure of materials with 10 wt% of SiC additions (0.2–0.8 μm) prepared from Mg(I):2B(I) under 2 GPa (HP) at 800 °C for 1 h (**a**–**d**) and at 1050 °C (**e**–**h**); (**a**,**c**,**e**,**g**)—SEI images; (**b**,**d**,**f**,**h**)—COMPO images; (**a**,**b**), (**c**,**d**), (**e**,**f**), and (**g**,**h**) are paired images of the same place under the same magnification but in different modes—SEI and COMPO [132].

The structures of magnesium diboride synthesized with additions of SiC (200–800 nm grain sizes), under 2 GPa at 800 and 1050 °C for 1 h from Mg(I):2B(I), are shown in Figure 17a–h [20,125,126]. The sample synthesized at 1050 °C has the highest critical current density reported in the literature (Figure 10c). The X-ray study did not find a visible interaction between MgB_2_ and SiC, and also found the formation of Mg_2_Si (Figure 17). The addition of SiC, like in the case of Ti, promotes the impurity of oxygen for aggregation into separate inclusions, even at 800 °C (the brightest small inclusions in Figure 17c). The superconducting characteristics of the HP-synthesized MgB_2_ samples in which Mg_2_Si is detected by X-ray are not so high, and sometimes even lower than those of the materials without additions, which indicate that overdoping with carbon is not useful. The interesting fact is that SiC additions improve *J*_c_ if the initial boron contains the smallest amount of an admixture of oxygen (Figure 10c), but are not effective when the boron contains a higher amount of an admixture of oxygen. In the case of Ti additions, it is vice versa. It has been assumed that nanosized grains of SiC can act as pinning centers in the MgB_2_ matrix [41,42,43,44]. The oxygen-enriched Mg-B-O inclusions are invisible on the image obtained by SEM in the COMPO regime (Figure 17h), but are very well seen in SEI mode, as the brightest small inclusions in Figure 17g. And, vice versa, the SiC inclusions are very well seen in the COMPO regime and are not so bright in SEI mode. Thus, using SEM SEI and COMPO modes, the inclusions of SiC and Mg-B-O can be revealed in the MgB_2_ matrix.

Some SiC grains are agglomerated, but some of them are rather small. The boundaries of the SiC grains can play the role of additional pinning centers. The SiC additions also affect the agglomeration of an admixture of oxygen into separate inclusions, even at low synthesis temperatures. As in the case of a Ti addition (Figure 10d), the mechanism of the positive effect of SiC additions on *J*_c_ (Figure 10c) is not fully understood yet.

### 4.3. Effect of Ti-O and TiC Additions

**Figure 18:**
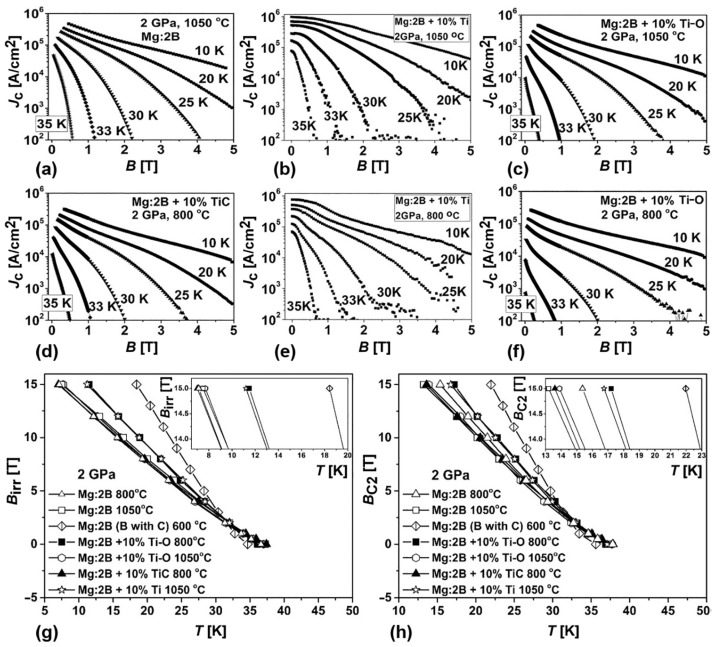
Characteristics of MgB_2_-based materials synthesized from Mg(I):2B(III) and Mg(II):2B(II) under 2 GPa for 1 h at different temperatures: (**a**–**f**)—dependences of critical current density, *J*_c_, on magnetic field, *B*, of materials without (**a**) and with additions of titanium (Ti) (**b**,**e**), polyvalent titanium oxides (Ti-O) (**c**,**f**), and titanium carbide (TiC) (**d**); (**g**)—fields of irreversibility, *B*_irr_, and (**h**) upper critical magnetic fields, *B*_C2_, vs. temperature [85].

The effect of Ti-O and TiC additions on the superconducting properties of MgB_2_ superconductors prepared under HP conditions has been studied by the authors of [85]. Figure 18 presents the magnetic field dependence of the critical current density, *J*_c_, and the temperature dependences of the irreversibility, *B*_irr_, and upper critical, *B*_C2_, magnetic fields of MgB_2_ materials, both without and with additions of TiC and Ti-O. For comparison, the characteristics of the material prepared from Mg(I):2B(III) with Ti additions are also presented. In Figure 18g,h, the temperature dependences of *B*_C2_ and *B*_irr_ of the superconductors prepared using HyperTech produced boron (B(II)) and fine Mg(I), with the specially added carbon (3.5 wt%) also shown. The sample with a 10% Ti addition prepared under 2 GPa at 1050 °C has the highest critical current density in a magnetic field of 1–5 T (Figure 18b). Despite the critical current density, *J*_c_, of the MgB_2_-Ti-O synthesized at 800 °C being lower than those of the MgB_2_, MgB_2_-Ti, and MgB_2_-Ti-O samples synthesized at 1050 °C (Figure 18a–f), its magnetic fields, *B*_irr_ and *B*_C2_, are higher (Figure 18g,h). The MgB_2_-TiC sample synthesized at 800 °C has an upper critical magnetic field about equal to that of the samples without additions prepared at 800 and 1050 °C. The irreversibility field, *B*_irr_, of the MgB_2_-TiC is lower than of the MgB_2_ prepared at 800 °C. Table 7 presents the results of the study of connectivity, *A*_F_, shielding fraction, *S*, and transition temperature, *T*_c_ [85]. All the materials have a shielding fraction of 86–100%, but their connectivities are rather different.

Thus, a connectivity near 80% is demonstrated by the materials without additions prepared at 800 °C and 1050 °C. The materials with Ti additions have the highest critical current density, *J*_c_, in fields up to 4 T (Figure 18b,e), but their connectivity is lower than that of the materials without additions synthesized at the same temperatures (Table 7). The MgB_2_-TiC sample has a somewhat lower connectivity than that of the materials with Ti additions. The MgB_2_-TiC critical temperature, *T*_c_, is the highest (Table 7), but its critical current density, *J*_c_, at 1–5 T is the least (Figure 18). The lowest connectivity, but the highest magnetic fields, *B*_C2_ and *B*_irr_, are demonstrated by the MgB_2_-Ti-O sample synthesized at 800 °C. All the materials studied in [85] were prepared from the same initial B(III) and Mg(I). The variations in the compositions of the material structures are shown in Table 8. The matrices of MgB_2_ contain less impurity of oxygen than theMg-B-O inclusions, and no carbon in the case of the Ti-O addition, opposite to the case of the TiC addition (Table 8). The inclusions of Ti-O absorb (or react with Mg) a rather high amount of Mg and some small amount of carbon (Table 8).

## 5. Structure of Superconducting Magnesium Diboride and Substitution of Boron Atoms by Oxygen and Carbon

The typical structure of MgB_2_ materials synthesized at low (800 °C) and high (1050 °C) temperatures under 2 GPa are shown in Figure 9. As established in [20,56,78,79,87], the structure changes caused by a synthesis temperature increase are schematically shown in Figure 9c,d. An X-ray analysis of MgB_2_-based materials synthesized at 1050 °C shows that they contain MgB_2_ and MgO phases (Figure 9e,f). However, SEM and EDX analyses and an Auger spectroscopy study indicate the presence of three main phases in the materials (Figure 9): (1) a matrix with near–MgB_2_ stoichiometry, which contains a small amount of impurity of oxygen (grey areas in the photo); (2) inclusions (grains) of higher magnesium borides, MgB_x_, with x >> 2 looking the blackest; (3) nanolayers (if the synthesis temperature was low) or separate oxygen–enriched inclusions (if the temperature was higher) with a stoichiometry close to MgBO (oxygen–enriched places look the brightest or white) [108].

The possibility of impurities or specially added carbon atoms replacing boron atoms in MgB_2_ is well known. The results of an Auger study and Rietveld refinement of the X-ray patterns of the materials with high critical current densities show that a small amount of oxygen, 0.2–0.32 atoms per one unit cell of MgB_2_, are present in all the studied materials.

To analyze the existence of Mg, B, and O elements, a quantitative Auger analysis of the depth of the MgB_2_ material matrix or so-called “depth profile” was used in [85]. The quantity of elements was estimated in the same place of the structure (marked by a white cross in Figure 9b) after each of multiple etchings by Ar ions in the chamber of a microscope. The Auger analysis shows that the MgB_2_ matrix phase contains some amount of oxygen, and the stoichiometry of the phase containing oxygen is about MgB_2.2–1.7_O_0.4–0.6._ The set of quantitative Auger tests was performed up to a depth of 200–300 nm. The Auger spectra indicate the presence of a constant amount of oxygen in the MgB_2_ matrix that, in turn, can witness about the formation of solid solutions of oxygen in MgB_2_.

These facts stimulated the authors of [10,11,117,130,132] to perform detailed structural studies of MgB_2_ and modeling of electron density in MgB_2-x_Ox structures, binding energy, structure variations, and enthalpy of solid solutions formation.

Rietveld refinements of the MgB_2_ phases of the X-ray patterns of 10 samples with high critical current densities have demonstrated that they contained some solved oxygen, the amount of which was very similar in all the materials—within MgB_1.68–1.8_O_0.2–0.32_ stoichometry [10,11,117,130].

The results of ab-initio modeling have shown that the replacement of boron atoms with oxygen is energetically favorable if oxygen is substituted for boron up to the composition MgB_1.75_O_0.25_ (The enthalpy of MgB_2_ and MgB_1.75_O_0.25_ formation were estimated as ΔH_f_ = −150.6 meV/atom and Δ*H*_f_ = −191.4 meV/atom, respectively).

In the case of carbon substitution, even very small levels of doping can essentially affect the superconducting characteristics of a material, due to changing its electron density. However, if oxygen substitutes for boron (especially in nearby positions of the same boron layer in a MgB_2_ unit cell), the substitution slowly changes the superconductive properties of MgB_2_. The formation of vacancies at the Mg site in both the MgB_2_ and MgB_1.75_O_0.25_ phases has also been modeled. However, it was found that this vacancy formation is energetically disadvantageous. It was estimated by the authors of [87] that ΔH_f_ of Mg_0.875_B_2_ and Mg_0.75_Ba_1.75_O_0.25_ are equal to −45.5 and −93.5 meV/atom, respectively.

**Figure 19:**
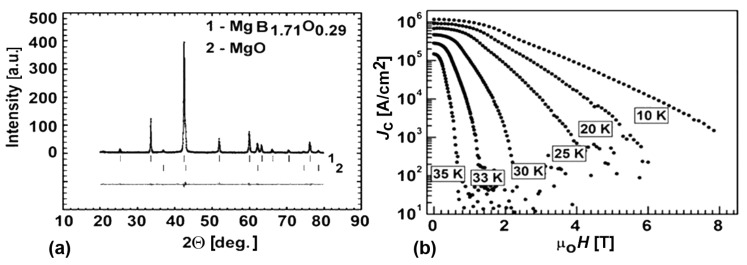
(**a**)—X-ray diffraction pattern, (**b**)—dependence of critical current densities, *J*_c_, on magnetic field, µ_o_*H*, at 10, 20, 25, 30, 33, and 35 K of the material, prepared from Mg(I):2B(I) under 2 GPa at 1050 °C for 1 h [117].

The X-ray study of MgB_2_ prepared from Mg(I):2B(I) under 2GPa at 1050 °C for 1 h demonstrates that MgB_1.71_O_0.29_ and MgO are (Figure 19) the structure of the main matrix phase.

The dependence of the critical current density of the sample on temperature and magnetic field is shown in Figure 19b.

The various theoretical aspects of MgB_2_ have been considered in many publications (e.g., [70,72,81,85,140] and the references therein). Here, we briefly discuss the recently obtained results of the calculation of the electronic states in MgB_2_.

**Figure 20:**
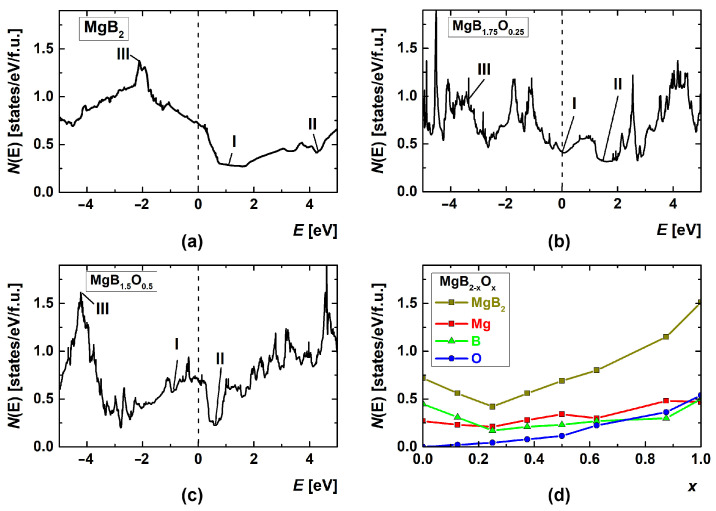
Calculated density of electronic states, *N*(*E*), for MgB_2_ (**a**), MgB_1.75_O_0.25_ (**b**), MgB_1.5_O_0.5_ (**c**) per formula unit; (**d**)—calculated DOS at the Fermi level. *N*(*E*_F_) depends on the oxygen concentration, x, in MgB_2-x_O_x_ compounds (hollow squares). The total DOS and partial contributions of Mg, B, and O atoms are indicated by solid squares, solid triangles, and solid circles, respectively [132].

Calculations of the density of electronic states *N*(*E*) (DOS) for different concentrations of oxygen substituting for boron were performed in [132], which assumed that the oxygen atoms were in the same positions as the substituted boron ones. The authors of [132] found changes in the positions of the *N*(*E*) peaks, marked I, II, and III in Figure 20. The calculated DOS *N*(*E*) for the Mg-B-O supercells revealed significant hybridization of the *s* and *p* states of Mg, B, and O. With an increase in the oxygen content, x, in MgB_2-x_O_x_, the hybridization of the Mg, B, and O states ensures an increase in the DOS *N*(*E*) near-Fermi level, *E*_F_ (Figure 20d). An increase in *N*(*E*_F_) with the oxygen concentration (x→1) leads to an increase in the total energy, and the minimum of free energy cannot be realized. This may explain the appearance of separate oxygen-enriched inclusions with increasing oxygen concentration, such as MgO and Mg-B-O [132].

The calculations of the DOS for MgB_2-x_O_x_ and MgB_2-x_C_x_ compounds for 0 < x ≤ 1 demonstrate that all the compounds have a metal-like behavior near the Fermi level [130]. In the case of the substitution of boron by oxygen, the lowest DOS of about 0.46 states/eV/f.u. is found for MgB_1.75_O_0.25_, if the oxygen atoms are in neighboring positions [130]. The calculations show that the MgB_2_ structure is destroyed if the concentration of oxygen is higher than that in MgB_1.5_O_0.5_. The lowest DOS of about 0.3 states/eV/f.u is found for MgB_1.5_C_0.5_.

The modeling of the electron localization function (ELF) for MgB_2_ and MgB_1.75_O_0.25_ allowed the authors of [117] to conclude that the higher electron concentration in MgB_2_ is between the boron atoms and corresponds to strong covalent bonding within the boron network. In the places where boron atoms are substituted by oxygen ones, the electrons localize around the oxygen atoms and, thus, bonding polarization appears. The variation in ELF occurs because oxygen atoms affect nearby B-B bonds and B-O bonds.

**Figure 21:**
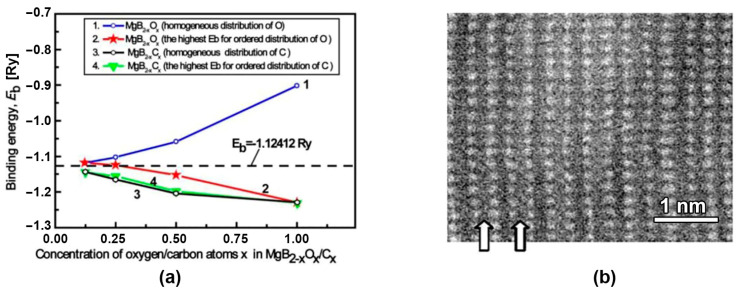
(**a**)—Dependence of the binding energy, *E*_b_, on the oxygen concentration, x, in MgB_2-x_O_x_/C_x_: 1, 3—homogeneous oxygen and carbon substitutions of boron atoms, respectively; 2, 4—the lowest binding energy vs. x for the ordered oxygen and carbon substitutions (for example, in nearby positions or in pairs), respectively. (**b**)—Z-contrast image of coherent oxygen-containing inclusions in [010] of MgB_2_ obtained using HRTEM (high–resolution transmission microscopy). Bright atoms—Mg. The contrast increases in each second row and is due to the presence of oxygen in each second boron plane. The white arrows show the columns of atoms in which oxygen is present [117].

Figure 21a shows the dependence of binding energies, *E*_b_, calculated using WIEN2k on the boron/oxygen/carbon concentration, x, in MgB_2-x_O_x_/C_x_, when oxygen and/or carbon substitute for boron in MgB_2_ randomly (homogeneously) and in ordered (nearby) positions [117,132]. The lowest binding energy, *E*_b_, for each concentration of oxygen atoms distributed in a certain order is shown in Figure 21a, curve 2, and for when they are distributed homogeneously—in Figure 21a, curve 1.

**Figure 22:**
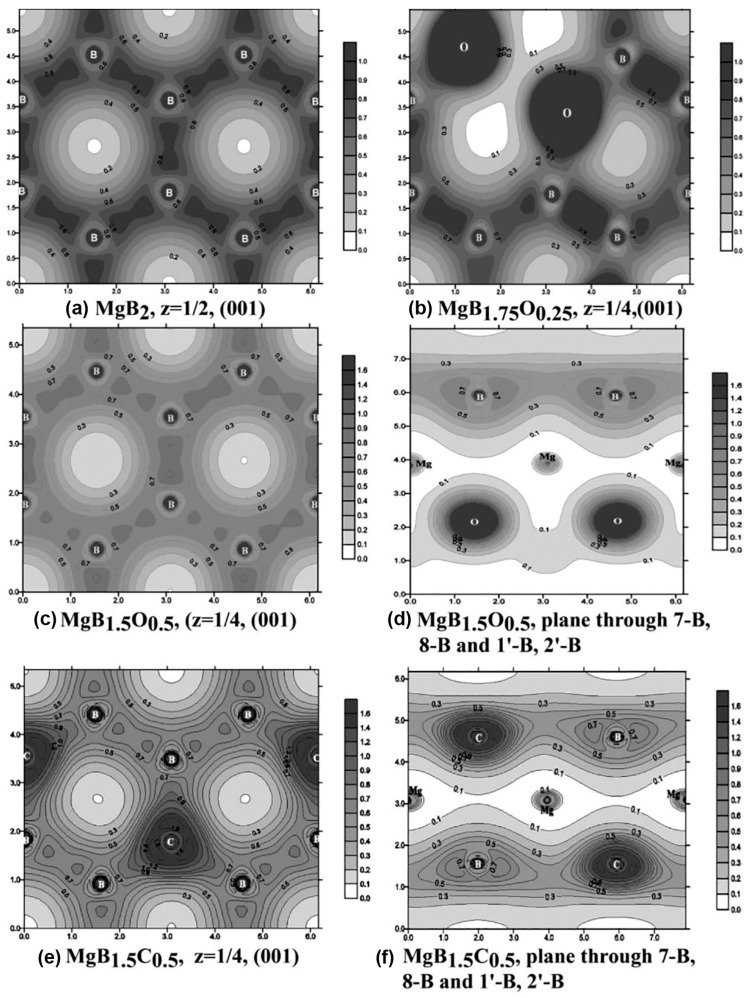
Maps of electron density distribution for: (**a**)—MgB_2_ (z = 1/2, (001)), (**b**)—MgB_1.75_O_0.25_ (z = 1/4, (001) [108]), (**c**)—MgB_1.5_O_0.5_ (z = 1/4, (001)); z-coordinates of the plane of a 2 × 2 × 2 supercell, where z is given in units of the *c* parameter of a 2 × 2 × 2 MgB_2_ supercell [132]; (**d**)—MgB_1.5_O_0.5_ in the transversal plane under an angle to the basal boron planes of the hexagonal unit cell to show the boron plane without imbedded oxygen atoms together with the Mg plane (the plane goes through the 7-B, 8-B, and 1′-B, 2′-B positions of a 2 × 2 × 2 supercell [132]); (**e**)—MgB_1.5_C_0.5_ (z = 1/4, (001)); (**f**)—MgB_1.5_C_0.5_ in the transversal plane under an angle to the basal boron planes (the plane goes through the 7-B, 8-B, and 1′-B, 2′-B positions of a 2 × 2 × 2 supercell) [117].

The maps of the electronic density distributions of the MgB_2,_ MgB_2-x_O_x_, and MgB_2–0.5_C_0.5_ structures are shown in Figure 22. Figure 22b shows the boron plane with the embedded oxygen atoms in nearby positions when oxygen atoms are absent in the second (alternate) boron plane of the same unit cell (Figure 22c). Figure 22d displays a cut of the unit cell inclined to the basal boron planes, displaying two boron planes. The top plane contains only boron atoms; some boron atoms are substituted by oxygen in the bottom plane (Figure 22d). If oxygen moves into nearby boron positions or forms even zigzag chains the lowest *E*_b_ is obtained. This can explain the following effects: the tendency of oxygen aggregation in the MgB_2_ structure, the formation of oxygen-enriched layers or inclusions, and a rather high amount of oxygen can be present in superconducting MgB_2_ with a higher transition temperature.

The Z-contrast image of the coherent oxygen-containing inclusions in the MgB_2_ [010] bulk material is shown in Figure 21b. This image was obtained experimentally by the authors of [141] and shows that oxygen (if its amount is small) prefers to substitute for boron atoms in the second boron plane of each MgB_2_ unit cell, leaving the first boron plane pristine.

Figure 22e presents the boron plane of the MgB_1.5_C_0.5_ compound with the embedded carbon atoms, the binding energy of which is least according to the ab-inito calculations. Figure 22f shows the cuts of the unit cell MgB_1.5_C_0.5_, made in such a way as to show the boron plane with the Mg and C atoms.

If carbon is substituted for boron, the binding energy, *E*_b_, is about the same for the definite order (Figure 21a, curve 4) and homogeneous (Figure 21a, curve 3) distributions. Despite there being no difference from the energetic point of view as to whether carbon atoms substitute for boron ones in a special order or homogeneously, the embedding of carbon into the MgB_2_ structure can essentially decrease the critical temperature and critical current density, especially in low magnetic fields at relatively high temperatures.

## 6. Application of Bulk MgB_2_ Superconductors

Since the discovery of HTS and MgB_2_ bulk superconductors, they have competed with long wires and tapes for possible and real applications, such as small and middle power motors, shields, and the creation of DC magnetic fields [142,143]. For example, bulk superconductors can trap magnetic fields of an order higher than those trapped by permanent magnets (e.g., a trapped magnetic field can be of 5.4 T in bulk MgB_2_, at 12 K and 5.6 T at 11 K [144]). In addition, for the manufacturing of wires/tapes and thin films, a complex multi-step processing technique is required. Bulk MgB_2_ can be fabricated using an essentially simpler process. Unlike conventional magnets, a bulk superconductor magnet may be safely and conveniently demagnetized by simply heating above the critical temperature. The HTS-bulk prototypes of various devices have been designed and described in [143,145,146,147]. The operation principles of superconducting devices are independent of the superconductor type, and the choice of the type depends on the required superconducting properties, operation temperature, etc. The MgB_2_ superconductors with a bulk density of about 2.63 g/cm^3^ are the lightest materials among practical superconductors. This makes MgB_2_ attractive for portable applications [143,144,145,146,147,148,149,150,151,152,153,154,155,156,157,158,159,160,161,162,163,164,165,166,167,168,169,170,171,172,173,174,175,176,177,178,179], especially for aviation and space technology [26,146,171].

Here, we briefly consider some applications of MgB_2_ bulk materials.

**Figure 23:**
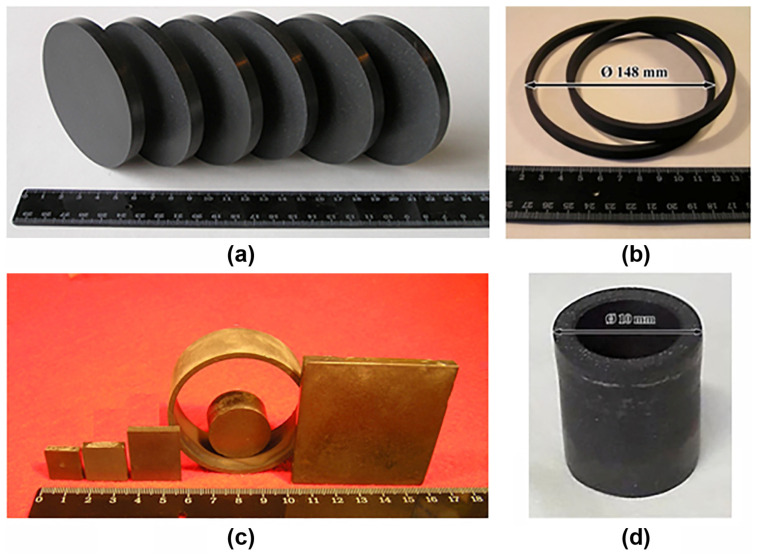
Examples of MgB2 bulk superconductors: (**a**)—obtained using HotP, (**b**) [120], (**c**) —obtained using HP and then the rings were cut mechanically, and (**d**)—obtained by machining a bulk cylinder manufactured using SPS [26].

The MgB_2_ bulk samples we are fabricated in the form of cylinders, cylinders with a bottom (cap), discs, and parallelepipeds (Figure 23) by different methods (hot pressing, high pressing, and spark plasma sintering). From these samples, rings and hollow cylinders were cut out by electro-erosion in oil [142] or in deionized water for the design of fault current limiter models, magnetic shields, etc.

**Figure 24:**
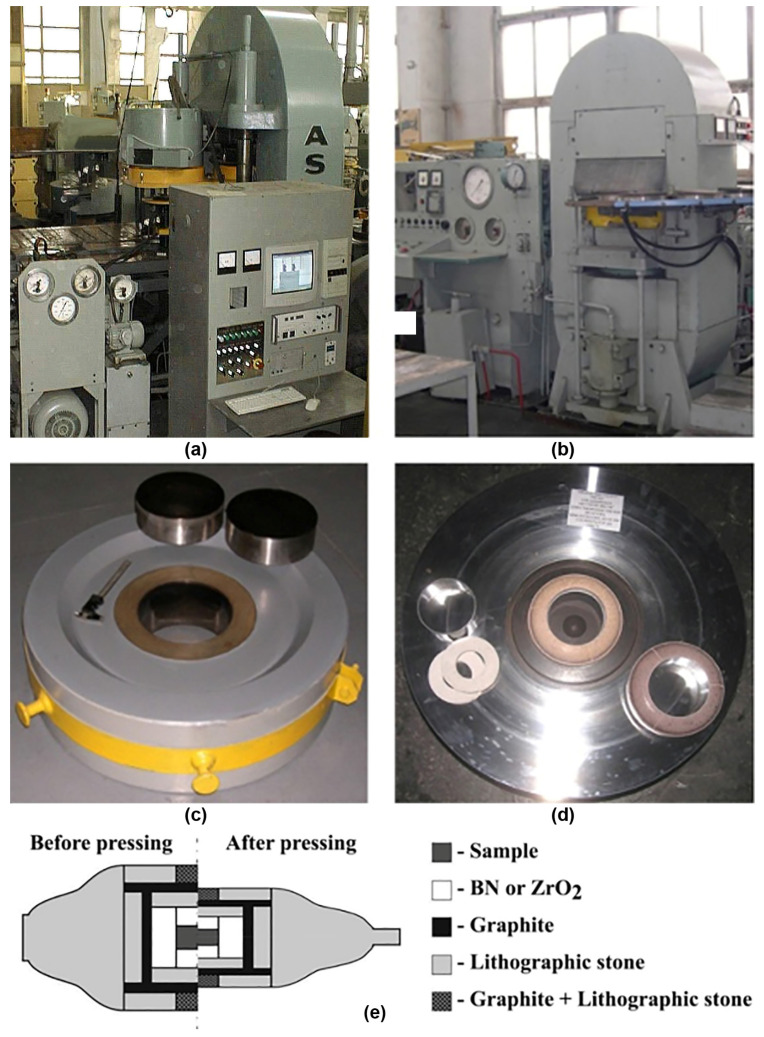
High quasi-hydrostatic pressing (HP) in ISM NASU. Hydraulic 140 MN-effort press from the ASEA company (**a**), hydraulic 25 MN-effort press (**b**), cylinder piston high–pressure apparatus (HPA) (**c**), recessed-anvil type (HPA) for 25 MN press (**d**), and scheme of high–pressure cell of the recessed-anvil HPA (before and after loading) (**e**).

**Figure 25:**
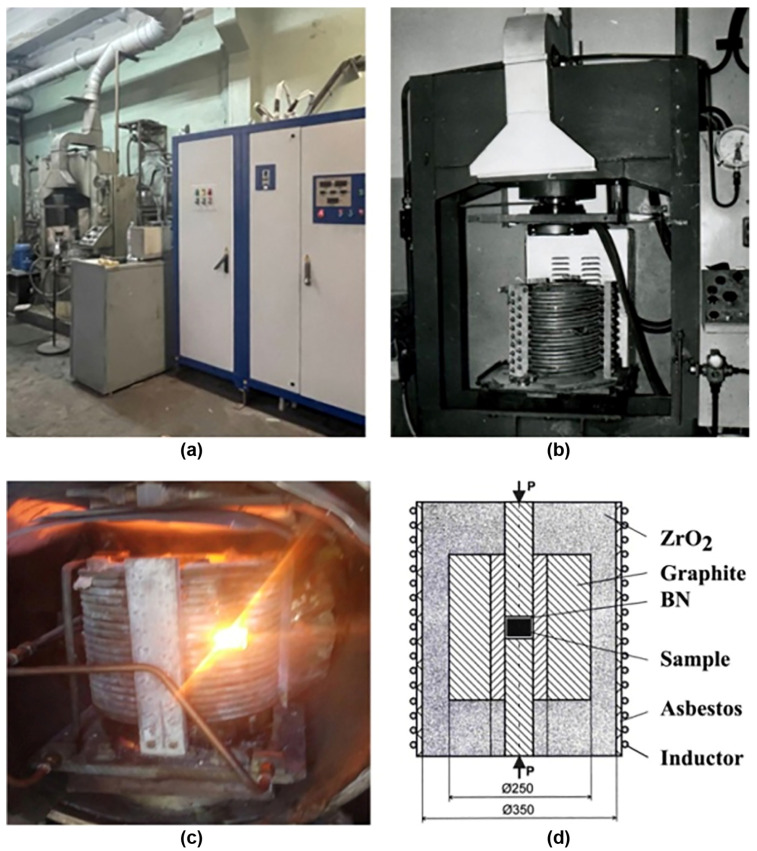
Hydraulic press DO 630 for hot pressing with generator and inductor (**a**,**b**); general view of inductor of hot press during heating (shining window—opening for temperature estimation by pyrometer) (**c**), scheme of assembled inductor (**d**).

**Figure 26:**
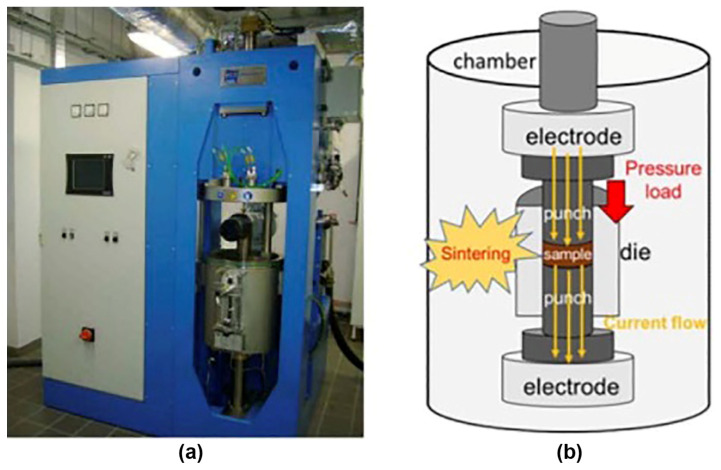
Installation for spark plasma sintering (**a**) and, scheme of SPS heating chamber (**b**) [166].

Figure 24, Figure 25 and Figure 26 show the typical equipment for the manufacturing of bulk MgB_2_ materials by different methods. The high–pressing (Figure 24), hot–pressing (Figure 25), and spark plasma sintering (Figure 26) equipment allow for manufacturing rather big blocks, the sizes of which are suitable for practical applications (up to 100–250 mm in diameter) with high critical currents, and are highly dense and mechanically stable. During the synthesis or sintering of magnesium diboride using these methods, MgB_2_ can be in contact with hexagonal boron nitride or with graphite stripe.

The method of high isostatic pressing (HIP) at a high temperature allows for the manufacturing of bulk materials with high superconducting characteristics as well, but needs encapsulation to be densified. The capsule should be hermetized and soft enough under a high temperature to transmit gas pressure toward the green body of the sample or block, and be inert toward magnesium diboride. The HIP equipment for a big volume is rather unique and complicated.

### 6.1. Trapped Magnetic Field (Quasi-Permanent Magnets)

Magnetized MgB_2_ and HTS bulks can be used as quasi-permanent magnets providing magnetic fields of several Tesla or even more than ten. These values are much (up to an order) higher than a magnetic field, which can provide the best traditional permanent magnets. This opens a way to apply these superconductors as permanent magnets in various devices, such as flywheel energy storage systems.

MT-YBCO bulks have demonstrated the possibility of trapping magnetic fields of 17.24 T at 29 K in the center of two 26 mm diameter samples impregnated with Wood’s metal and resin and reinforced with carbon fiber [148]. However, around 26 K [149] these reinforced samples have cracked. The trapped field of 5.4 T was measured in bulk MgB_2_ at 12 K on the surface of a single cylinder (20 mm diameter), fabricated by hot pressing of ball-milled Mg and B powders [144]. A uniaxial stack of two hot-pressed MgB_2_ disc-shaped bulk superconductors with a diameter of 25 mm and a thickness of 5.4 mm can trap 3.14 T at 17.5 K [150].

The trapped field of REBCO magnets is limited by the mechanical properties of the superconductors. The Lorentz force can be so high that samples can be destroyed. MgB_2_ bulk materials have demonstrated trapped fields higher than 3 T, although the trapped fields of MgB_2_ are less than those of MT-YBCO at 20 K. The advantage of MgB_2_ superconductors is that their preparation methods are much easier, cheaper, and quicker.

For many applications, several rings can be stacked to form the required experimental structure. For example, a three-ring stack can trap a field of 2.04 T at 20 K [159] and block (D30 × h7.5 mm). A structure synthesized from Mg(I):2B (V) with 10% Ti under 2 GPa, at 900 °C for 1 h traps a field of 1.8 T at 20 K [20].

All the methods noted above open a way to use bulk MgB_2_ superconductors as an element of the setup for physical experiments, medical devices, flywheel energy storage systems, levitation systems, electrical machines, etc.

### 6.2. Fault Current Limiters

The application of fast-operating nonlinear fault current limiters (FCLs) thet allow for the limiting of high fault currents due to the capability of increasing their impedance rapidly could be a promising solution to the fault current problem in power systems. Two properties of superconducting materials are the bases of SFCLs: an ideal conductivity in the superconducting state and a fast phase transition from this state into the normal conducting state with an increase in the current, magnetic field, or temperature above their critical values. SFCLs are one of the most attractive applications of superconductors in power systems, and there have been no classical equivalents up to now [120,136,145,146]. These devices meet all the power system requirements; this has been confirmed experimentally by testing models, prototypes, and experimental power devices of various types of SFCLs, based on different superconductors.

**Figure 27:**
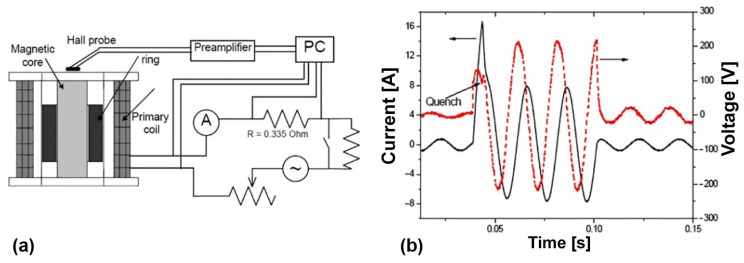
(**a**)—The schemes of an SFCL model and a testing circuit for the simulation of a fault event. (**b**)—Typical oscilloscope traces of the current in a protected circuit (black, solid curve) and the voltage drop across the primary coil of the SFCL model (red, dashed curve) at 50 Hz and about 4 K (from [90]). The experiment details are described in [120]. “A”—is ammeter.

Bulk MgB_2_ rings and hollow cylinders can be applied as active superconducting elements of inductive SFCLs. The principal inductive SFLC design and experimental set-up for SFCL model testing are presented in Figure 27a. Under the nominal regime of a protected AC circuit, the impedance of the SFCL, the primary coil of which is connected in series,, is low. During a fault event, the current in the circuit increases, causing a phase transition in the secondary superconducting coil, accompanied by an increase in the device impedance and, following that, a fault current limitation [145,146,151]. An inductive SFCL can be also used for the protection of high-voltage direct-current (HVDC) systems [152]. The secondary coil can be formed using a superconducting ring or a set of rings (hollow cylinders) to increase the SFCL power [145,146,151].

The character of the oscilloscope traces of the current in the circuit and the voltage drop across the primary coil of the inductive SFCL models is independent of the synthesis conditions and ring sizes. A low, long-continued current in the protected circuit (nominal regime) does not cause the transition of the superconducting ring into the resistive state. At a high current (simulates a fault event), the voltage and current curves’ deviations appear before the first current maximum (Figure 27b). These deviations are associated with the transition of the ring from the superconducting to the resistivity state, and with the quenching (critical) current of the ring. A set of FCL models with MgB_2_ rings prepared using various techniques and initial materials and additions has been built and successfully tested [91,120].

The sizes and synthesis conditions of the rings that have been tested as elements of an inductive SFCL are presented in Table 9. Note, that the experimental set-up for SFCL model testing (Figure 27a) can be used for measuring a “transport” critical current, AC losses, and voltage–current characteristics [120,151]. The “transport” critical current of the various rings was estimated as a quenching current, causing the transition. The highest value of 63,200 A/cm^2^ was obtained for Ring 3 (Table 9), with an outer diameter of 45 mm, a height of 11.6 mm, and wall thickness of 3.3 mm. The ring was prepared under a pressure of 30 MPa at 800 °C for 2 h. From the magnetization experiments, the critical temperature of these rings was estimated to be about 38 K.

The large difference between the critical current measurement results obtained by the two methods (Table 9) can be explained by:

- the granular MgB_2_ structure—the critical values are different for currents inside and between the granules;
- micro-cracks, which can play the role of centers of the normal zone nucleation;
- dynamic magnetic and thermal instabilities of the superconducting state.

### 6.3. Electrical Machines

The application of superconductors in electrical machines is mainly connected with replacing the traditional normal metal wires in the design with superconducting ones. Progress in the electromagnetic properties of bulk superconductors has opened a way to design other types of electrical machines with bulk–superconducting rotor elements (see, e.g., [145,154,155,156] and the references therein). It has been shown that these machines are effective in low and medium power ranges. Series prototypes of various types of machines (trapped field, hysteresis reluctance, etc.) have been designed using bulk YBCO superconducting elements and successfully tested in a wide temperature range. The authors of [104] presented the worldʹs-first motor (1.3 kW) built with a bulk high—pressure—high temperature—synthesized MgB_2_ superconductor. The superconducting elements of the reluctance motor rotor were made of MgB_2_—10 wt% Ti and synthesized under 2 GPa at 800 °C for 1 h.

**Figure 28:**
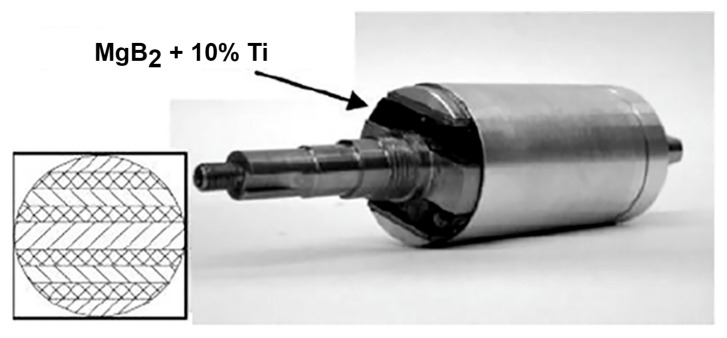
General view of azebra-type rotor of a 1300W/215V superconducting motor with MgB_2_ bulk superconductor [9].

Figure 28 demonstrates the general view of the zebra-type rotor (superconducting layers alternate with ferromagnetic ones) of a MgB_2_-10%Ti motor of 1300 W at 210–215 V. The comparative tests of the motor with MT-YBCO elements at the temperature for testing the MgB_2_ motor, 20 K, have shown that the efficiency of these motors is of the same level [19,20].

The integral part of hydrogen energetics would be systems for the production, salving, and transportation of liquid hydrogen [157]. Liquid hydrogen systems could be one of the first fields of application of MgB_2_ motors and submersible liquid hydrogen (LH) pumps. The small- and middle-power electrical motors based on MgB_2_ bulk superconductors have demonstrated efficiency higher than that of traditional motors and are cheaper than HTS motors. These pumps require superconducting magnets with trapped fields of around 500–600 mT. A bulk MgB_2_ superconductor is suitable for such applications at liquid hydrogenʹs temperature [142].

### 6.4. Magnetic Field Shields

Bulk MgB_2_ superconductors have shown excellent magnetic shielding properties [26,158,159,160] that can be useful for the passive shielding of various devices (measurement and medical devices, physical setup, etc.) and even for the protection of orbital stations in space from cosmic radiation. Also, the raw materials are largely available and do not contain rare earths, noble, or toxic elements, as in the case of other high- or low-temperature superconductors. In the literature, the results of the study of various designs of bulk MgB_2_ shields have been presented (e.g., [158,159], and the references therein).

**Figure 29:**
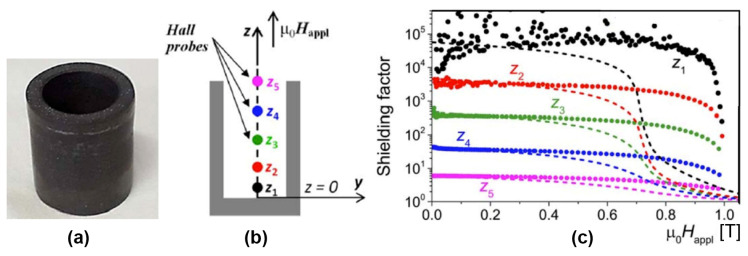
(**a**) Magnetic shield of MgB_2_ in the shape of a cup (outer radius, *R*_o_ = 10.15 mm; inner radius, *R*_i_ =7.0 mm; external height, *h*_e_ = 22.5 mm; internal depth, *d*_i_ = 18.3 mm). The material is machinable by chipping. The shielding factors (i.e., the ratio between an outer applied magnetic field, *H*_appl_, and an inner magnetic field measured by a Hall sensor at different z_1_–z_5_ positions (**b**)) at *T* = 30 K are shown in (**c**). The dashed lines represent the shielding factors computed in correspondence with the Hall probe positions, assuming the magnetic field dependence of *J*_c_(*B*) at 30 K. (Figure 2 in [26] adapts the results obtained in [159]).

As an example, the results of the magnetic shield properties of MgB_2_ bulk materials in the shape of a cup are considered. The experimental shielding factors (dots in Figure 29c) are practically independent of the applied field, up to ~0.8 T [26,159]. The factor strongly depends on the Hall probe position and reaches its maximum value, of the order of 10^5^, near the bottom of the cup. In the middle point, z_3_, the factor is ~250; this is sufficient in some cases.

## 7. Conclusions

This review examines the impact of technological parameters (pressure, temperature, etc.), additives, and impurities on the superconducting characteristics of MgB_2_-based bulk materials. The main attention is paid to the role of impurity oxygen in MgB_2_-based materials on the formation of their structures and on achieving the best superconducting characteristics (critical temperature and current density at 10–35 K in fields up to 10 T, temperature dependences of the upper critical, irreversibility, and trapped magnetic fields. The influence of additions of Ti, Ta, Zr, SiC, C, Dy_2_O_3_, Sn-O, Ti-O, TiC, and TiH_2_ with various production conditions on the structure (higher magnesium borides formation, oxygen and boron distributions, etc.) and superconducting properties is considered.

This analysis of publications, dedicated to studying the dependences of MgB_2_ bulk material properties on manufacturing pressure, presents the positive effect of a manufacturing pressure increase on superconducting characteristics. One of the main reasons for this improvement is the suppression of magnesium evaporation during the production process. This leads to an increase in the materialʹs density and connectivity between the superconducting grains.

The manufacturing temperature influences the dependence of the critical current density on magnetic fields: a higher manufacturing temperature results in higher critical currents in low magnetic fields, while a lower manufacturing temperature leads to higher critical currents in high magnetic fields. This effect is closely related to the oxygen admixture distribution: at higher manufacturing temperatures, separate oxygen-enriched inclusions appear, while oxygen-enriched nanolayers (or nanochains) form at lower manufacturing temperatures. 

Additionally, the variation of the critical current density can be connected with the formation and distribution of higher magnesium borides (x > 2) inclusions, observed in both in-situ (prepared from Mg and B) and ex-situ (prepared from MgB_2_ powder) materials. In the materials prepared at higher temperatures, the amount and size of inclusions of higher magnesium borides are smaller than in materials obtained at lower temperatures. These effects are more pronounced for materials produced at high pressures (2 GPa).

It was shown that superconducting materials with high magnetic properties can be obtained with even a large deviation from the MgB_2_ composition (initial Mg:4B–Mg:20B mixtures). 

In MgB_2_ superconducting materials exhibiting extremely high critical current densities, the dissolutions of a small amount of oxygen and the formation of a superconducting matrix phase MgB_1.8–1.68_ O_0.2–0.32_ have been detected using X-ray analysis. Similar results were obtained using quantitative Auger analysis: matrix phases of MgB_2_ samples with high superconducting characteristics contain a small amount of impurity oxygen.

Modeling the structure of MgB_2-x_O_x_ solutions showed that the AlB_2_ structure type can be maintained even at x about 0.5. It was also shown, that the enthalpy of MgB_1.75_O_0.25_ formation is lower than that of MgB_2_ where oxygen replaces boron in nearby positions and penetrates only into one boron layer of the MgB_2_ cell. At the same time, the second MgB_2_ layer of the same cell remains intact, i.e., every second boron layer of the cell contains only boron atoms. This structure was observed in MgB_2_-based material using a High-Resolution Transmission Microscope.

Ti, Zr, Ta, Ti-O, and SiC additions can lead to impurity oxygen aggregation into separate inclusions at low manufactured temperatures; thus, the MgB_2_ matrix is “cleaned” from impurity oxygen or by reducing the volume that the Mg-B-O phase, containing a high amount of oxygen, occupies. Ti, Zr, and Ta additions are the absorbers of gases (e.g., hydrogen), and Ti is the most powerful one. So, they absorb the admixture of hydrogen transforming into hydrides and, thus, prevent the formation of the MgH_2_ phase that is harmful for critical currents. The absorption of hydrogen can prevent big blocks of MgB_2_–based superconductors from cracking. The presence of Ti and Ta “provokes” the appearance of inclusions of higher magnesium borides in higher amounts, which increases the critical currents in high magnetic fields. The effect of SiC on oxygen aggregation in MgB_2_ is not clear yet. The added, nanosized SiC inclusions can act as pinning centers in MgB_2_. However, SiC can partly decompose and react with the synthesized material forming Mg_2_Si and liberating C, which may be introduced into the MgB_2_ structure, forming a solid solution. The addition of SiC (10 wt %) with micrometer—sized grains, which practically do not react with MgB_2_ (at least in an amount detectable by X-ray), essentially increases the critical current density of the materials prepared from boron with a low concentration of impurity oxygen. The optimal level of carbon doping, without an essential reduction in the critical temperature of MgB_2_, is much lower than that for oxygen doping, regardless of whether carbon is homogeneously distributed or concentrated in the nearby positions. Modern technologies and high-pressure equipment allow for the manufacturing of superconducting MgB_2_ blocks of sizes suitable for the design of real devices, such as fault current limiters, magnets, magnetic bearings, pumps for liquid helium and hydrogen transportation, and electrical machines. This analysis of the published results shows that different production conditions and additions improve the superconducting properties of MgB_2_ bulks in various ranges of temperature and magnetic fields. That allows for choosing the optimal technology according to the application requirements. Bulk magnesium diboride superconductors are highly competitive and have several undeniable advantages over HTS bulk superconductors. Bulk superconductors can successfully compete with superconducting wires in the creation of magnetic shields (screens) and low- to medium-power devices.

## Figures and Tables

**Table 1:** Typical characteristics of initial boron and magnesium diboride powders and admixtures found in them. The data presented in the table were collected from [20,115,128].

Name	Amorphous Boron (B) Powder					Magnesium Diboride Powder (MgB_2_)			
Type	I	II	III	IV	V	VI	VII	VIII	IX
Average grain size	<5 μm	<1 μm	4 μm	0.8 μm	1.4 μm	9.6 μm	-	4.2 μm	9.0 μm
Purity, %	-	-	96.4	95.9–96.51	-	-	98	-	-
B, wt%	-	-	-	-	-	-	-	44.1	45.3
Mg, wt%	0.49	-	0.5	0.34–0.6		-	-	51.6	49.69
O, wt%	0.66	-	1.5	1.6–1.7	1.9	0.8	-	1.9	1.7
C, wt%	0.31 *	3.5 *	0.3 0.47 *	0.31 *	0.27 *	-	-	0.9	0.21
N, wt%	0.48 *	1.02 *	0.1 0.40 *	0.1–0.08 0.40 *	0.43 *	-	-	-	-
H, wt%	0.32 *	0.87 *	0.37 *	0.43 *	0.11 *	-	-	-	-
H_2_O, wt%	-	-	0.2	0.14–0.3	-	-	-	-	-
B-H_2_O, wt%	-	-	0.1	0.11–0.1	-	-	-	-	-
B-H_2_O_2_, wt%	-	-	0.5	0.3–0.48	-	-	-	-	-
Fe, ppm	-	-	-	-	-	-	-	0	165

Note: (1) The amounts of C, H, and N in the initial boron marked by asterisks (*) were obtained by using the Universal Micro Analyzer “vario MICRO cube” of the ELEMENTAR vario-analyzer family. (2) The manufacturing company provided information about the amount of oxygen, grain size, and carbon and nitrogen contents (which are not marked by asterisks). (3) The higher amount of C and N determined by the “vario MICRO cube” as compared to the producer’s estimation may be explained by chemical reactions during storage. (4) All “in-situ” materials were prepared from different types of amorphous boron using Mg(I) chips, and only samples from Type II boron with C addition were prepared using Mg(II) powder.

**Table 2:** Characteristics (*J*_c_, concentrations of MgB_2_, MgO, and MgB_4_; mass density, ρ; connectivity, *A*_F_; and amount of shielding fraction, *S*,) of MgB_2_-based materials prepared under different p–T conditions from Mg:2B mixtures (in-situ) or MgB_2_ powder (ex-situ). The data presented in the table were collected from [98,103,108,119].

No	Preparation	Type of B or MgB_2_	P [MPa]	T [°C]	*J*_c_ [MA/cm^2^], at 0–1 T, at 20 K	MgB_2_/MgO/MgB_4_ [wt%]	Density, ρ [%]	*A*_F_ [%]	*S* [%]
1.	in-situ, HP	I	2000	1050	0.9–0.7	94/6/0	99	-	-
2.	in-situ, HP	I	2000	800	0.2–0.15	91/5.5/0	98	-	-
3.	in-situ, HP	III	2000	1050	0.4–0.3	87/13/0	99	79	94
4.	in-situ, HP	III	2000	800	0.12–0.07	73/12/0	97	57	91–100
5.	in-situ, HP	II	2000	600	0.14–0.05	64/30/0	83	18	90
6.	in-situ, SPS	III	50	1050	0.5–0.45	83/4.5/12.5	94	98	91
7.	in-situ, SPS	III	50	800	0.4–0.36	-	74	-	-
8.	ex-situ, SPS	VII	50	1050	0.4–0.3	83/6.5/10.5	96	80	100
9.	in-situ, HotP	III	30	1050	0.08–0.016	46/8.5/45.5	99	32	-
10.	in-situ, HotP	III	30	800	0.3–0.2	-	72	73	-
11.	in-situ, PL	IV	0.1	800	0.08–0.03	90/10/0	55	-	-
12.	in-situ, PL, cold densified at 2 GPa	III	0.1	600	0.26–0.13	94.5/5.5/0	65	-	75

**Table 3:** Maximal pinning forces, *F*_p_(max), at 20 K and pinning types in MgB_2_-based superconducting materials manufactured under various pressure–temperature conditions from Mg:2B, both without and with different additions: SiC (10 wt%), Ti (10 wt%), and C (3.5 wt%), or from MgB_2_ powder [7].

Preparation, Addition, (Conditions)	Type of B	*P* [MPa]	*T* [°C]	*F*_p_(max)/ 10^9^ [N/m^3^]	*k* = *B*_peak_/*B*_n_	Pinning Type
in-situ, SiC (HP)	I	2000	1050	10.9	0.51	PP
in-situ, SiC (HP)	I	2000	800	1.9	0.31	GBP
in-situ, (HP)	I	2000	1050	7.6	0.53	PP
in-situ, (HP)	I	2000	800	1.6	0.36	GBP
in-situ, Ti, (HP)	III	2000	1050	4.8	0.42	MP
in-situ, Ti, (HP)	III	2000	800	1.9	0.24	GBP
in-situ, (HP)	III	2000	1050	2.3	0.43	MP
in-situ, (HP)	III	2000	800	0.8	0.30	GBP
ex-situ, (HP)	VIII	2000	1050	3.1	0.30	GBP
in-situ, (SPS)	III	50	1050	4.6	0.63	>PP *
ex-situ, (SPS)	VII	50	1050	3.3	0.58	>PP *
in-situ, (SPS)	III	50	800	2.7	0.56	>PP *
in-situ, C (HP)	II	2000	600	0.6	0.31	GBP
in-situ, Ti (HP)	III	30	1000	2.7	0.42	MP
ex-situ, (SPS)	VII	16	1150	1.5	0.45	PP
in-situ, (PL)	III	0.1	800	1.9	0.35	GBP

Note; All “in-situ” materials were prepared from Mg(I) chips and only C was added to initial boron and Mg(II) powder. PP, GBP, and MP—point, grain boundary, and mixed type of pinning, respectively. * Type of pinning is impossible to characterize exactly due to high *k* ratio.

**Table 4:** The critical current density, *J*_c_, and lattice parameters of the MgB_2_ phase vs. the average size of crystallites (grains) in the superconductor high-pressure sintered from MgB_2_ (VI) and synthesized from Mg(I):2B(III) [103].

HPS under 2 GPa for 1 h at *T*_s_ [°C]	Average Crystal Size	Lattice Parameters		*J*_c_, [kA/cm^2^] at 10 K		*J*_c_ [kA/cm^2^] at 20 K	
		*a* [nm]	*c* [nm]	0 T	1 T	0 T	1 T
From MgB_2_(VI) or ex-situ							
700	19.7 nm	0.30805	0.35188	Was not determined			
800	18.8 nm	0.30822	0.35212	Was not determined			
900	18.5 nm	0.30820	0.35208	56	14	36	8
1000	25.0 nm	0.30797	0.35200	28	8	19	6
From Mg (I) and B (III) in 1:2 ratio or in-situ							
800	15.0 nm	0.30747	0.35188	245	142	138	79
1000	37.0 nm	0.30808	0.35192	485	364	360	237

**Table 5:** Critical current density, *J*_c_, vs. relative amount, *N*, of inclusions with near-MgB_12_ stoichiometry of high-pressure samples synthesized from Mg(I) 2B(IV), both without and with additions of Ti and Ta [103].

Manufacturing Parameters: Pressure, *P*, Temperature, *T*, Holding Time, τ	Addition and Its Amount [wt%]	*J*_c_ 1 T, at 20 K [kA/cm^2^]	*N* [%]
*P* = 2 GPa, *T* = 800 °C, τ = 1 h	Ta, 10%	240	12.5
	Ti, 10%	360	14
	without	131	10.8

Here *N* is the ratio of the area occupied by the MgB_12_ inclusions in the COMPO image obtained at 1600× magnification to the total area of this image.

**Table 6:** Results of the quantitative Auger analysis [atomic %] made for the points marked by No. 1–6 in Figure 15c, and located at the boundary between the MgB_2_ and big (about 60 μm) Ti grains in the sample prepared under 2 GPa at 800 °C for 1 h. The sample was etched in Ar in a JAMP−9500F chamber before the study [113].

Element/ Point No	B	Ti	O	Mg	C
1	22.2	10.5	33.1	22.6	11.7
2	24.5	10.8	33.1	21.2	10.3
3	41.4	31.4	8.2	3.4	15.5
4	41.3	28.2	9.1	3.2	17.7
5	4.1	13,9	44.9	29.1	8.0
6	6.5	10.1	44.8	29.1	9.4

**Table 7:** Characteristics of MgB_2_-based materials with various additions [85]: connectivity, *A*_F_ [%], shielding fraction, *S* [%], transition temperature, *T*_c_ [K], and MgO (estimated by X-ray), [wt%] vs. composition.

Characteristic	Materials			
	MgB_2_	MgB_2_ + Ti	MgB_2_ + TiC	MgB_2_+ Ti-O
material synthesized at 800 °C, 2 GPa, 1 h				
*A*_F_	77	57	54.5	26
*S*	86	90	87	98
*T*_c_	37.56	36.4	37.2	36.2
MgO	-	-	13	25
material synthesized at 1050 °C, 2 GPa, 1 h				
*A*_F_	79	50	-	23
*S*	94	100	-	100
*T*_c_	-	37.0	-	37.0
MgO	-	-	13	21

**Table 8:** Results of quantitative EDX analysis [85].

Initial Mixture, Preparation	Matrix	Inclusions Look Most Dark	Grains of Additions
Mg:2B + (Ti-O), 2 GPa, 800 °C, 1 h	MgB_1.65_O_0.2_	MgB_9_O_0.37_	TiO_0.8_C_0.05_Mg0_0.16_
Mg:2B + (Ti-O), 2 GPa, 1050 °C, 1 h	MgB_2.6_O_0.1_	MgB_6.4_O_0.13_	TiO_0.3_ C_0.05_ Mg_0.02_– TiO_2.2_Mg_1.6_
Mg:2B + TiC, 2 GPa, 800 °C, 1 h	MgB_1.6_O_0.4_– MgB_1.8_O_0.5_C_0.13_	MgB_7.2_O_0.18_	TiC_0.63_Mg_0.02_

**Table 9:** Quenching current and current density of the rings tested using the SFCL model at 4.2–6 K and a primary current frequency of 50 Hz. The data presented in the table were collected from [120,153].

No	Ring Sizes, mm			Manufacturing Conditions *				Quenching Current [A]	Quenching Current Density, [A/cm^2^]
	Outer Diameter	Wall Thickness	Height	Pressure	Temperature [°C]	Time	Additions [wt%], Initial B		
1	24.3	3.2	7	2 GPa	1050	1 h	SiC, 10 B(III)	4500	21,000
2	24.3	3.2	7.7	2 GPa	1050	1 h	SiC, 10 B(III)	5600	22,700
3	45	3.3	11.6	30 MPa	800	2 h	B(III)	24,000	63,200
4	21.3	14.1	3.5	2 GPa	800	1 h	B(III)	9350	18,950
5	112	6	10	2 GPa	800	1 h	B(III)	9880	16,400
6	45	12	5	30 MPa	1050	1 h	B(III) Ti, 12	14,800	24,700

* The mixture of Mg(I) chips and amorphous B(III) powders were taken into Mg(I):2B(III) stoichiometry, then 200–800 nm SiC or 30 µm Ti granules of 95% purity were added.
